# Recovery Patterns: Longitudinal Cluster Analysis of Physical Function Following Abdominal Surgery

**DOI:** 10.1097/SLA.0000000000006671

**Published:** 2025-02-18

**Authors:** Daan J. Toben, Astrid de Wind, Eva van der Meij, Judith A.F. Huirne, Mark Hoogendoorn, Johannes R. Anema

**Affiliations:** * Amsterdam UMC Vrije Universiteit Amsterdam, Public and Occupational Health, De Boelelaan 1117, Amsterdam, The Netherlands; † Amsterdam Public Health research institute, Personalized Medicine, Amsterdam, The Netherlands; ‡ Amsterdam UMC University of Amsterdam, Public & Occupational Health, Meibergdreef 9, Amsterdam, The Netherlands; § Department of Gynaecology, NoordWest Ziekenhuisgroep, Alkmaar, The Netherlands; ∥ Department of Obstetrics and Gynaecology, Amsterdam UMC, Meibergdreef 9, Amsterdam, The Netherlands; ¶ Department of Computer Science, Vrije Universiteit Amsterdam, Amsterdam, The Netherlands

**Keywords:** perioperative care, Recovery, longitudinal cluster analysis, growth mixture modeling, K-medoids clustering

## Abstract

**Background:**

A rise in the proportion of day surgery has seen a concomitant increase in the proportion of patients recovering at home. Blended eHealth is well situated to provide this group with medical support and supervision. However, a data-driven description of the heterogeneity is missing.

**Objective:**

To identify clinically meaningful patterns of functional recovery following abdominal surgery and describe how the emergent patient characteristics differ between them.

**Methods:**

This was a secondary data analysis of 2 data sets collected through 2 previously conducted RCTs. We used k-medoids clustering and growth mixture modeling on the longitudinal patient-reported outcome measurement information system physical function t-scores of 649 patients. Differences in patient characteristics between the resultant clusters were identified through statistical tests.

**Results:**

Three clusters—fast, intermediate, and uneven recovery—were identified regardless of the data set or statistical technique. A fourth cluster—relapse—was identified by both statistical techniques but only in the presence of heavy surgery. The fifth and sixth clusters—low gain and high gain—were identified for both light and heavy surgery, but only through k-medoids clustering.

**Conclusions:**

Trajectories of physical function following abdominal surgery are heterogenous but distinct clinically meaningful patterns can be extracted. This classification may facilitate shared decision-making during preoperative care, and future research may utilize them as targets for prediction.

Day surgery, defined as admittance to and discharge from the hospital within 24 hours of surgery, is increasingly implemented across Europe. This trend is driven by advancements in medicine,^1,2^ financial considerations,^3^ and patient preferences.^4^ As a consequence, an increasing number of patients recover at home rather than in the hospital.^5^ Patient experiences with recovery at home are mixed. Studies report feelings of insecurity, moderated through timely provision of information, expectation management, and professional support.^6–9^

Blended eHealth interventions, which combine health care with Information and Communication Technologies (ICT), may effectively address these needs. In the Netherlands, the mHealth intervention ikHerstel was developed with this purpose in mind. It supports patients’ postoperative recovery by providing them convalescence plans that help them regain physical functioning (eg, kneeling or riding a bicycle).^10–12^ Research has shown that IkHerstel accelerates recovery, reduces pain, and improves health-related quality of life for patients undergoing abdominal surgery.^13–16^ The app is implemented at the hospital level, where clinicians introduce patients to ikHerstel prior to surgery. Its implementation currently covers close to 10% of Dutch hospitals.^17^ However, individual variations in recovery speeds among patients using ikHerstel have been observed, indicating a lack of personalization in the recovery plans provided.^18^ This is a common weakness for mHealth applications, and in the case of ikHerstel, it may lead to suboptimal recovery, longer recovery times, increased pain, and reduced health-related quality of life.^19^

To address this issue, we aim to personalize ikHerstel’s convalescence plans by identifying clinically meaningful patterns of functional recovery and describing how patient characteristics differ between them. The resultant roadmap may help health care professionals (HCPs) remotely monitor patients’ progress and identify issues such as recovery stagnation or relapse. While similar work has been done for ankle surgery patients,^20^ abdominal types of surgery are lacking in this regard to the knowledge of the authors. We used data from 2 randomized controlled trials (RCTs) previously conducted within our research group.^15,16^ Our research question is: What patterns of functional recovery do patients recovering from abdominal surgery in the Netherlands exhibit? Our secondary question is: Which patient characteristics typify these patterns?

## METHODS

### Study Design, Data, and Setting

We employed secondary data analysis to cluster patients’ physical functioning following abdominal surgery. Data were sourced from two multicenter, single-blind, randomized, placebo-controlled trials,^15,16^ registered in the Netherlands National Trial Register (NTR4699 and NTR5686). The study protocol was preregistered on the Open Science Framework [https://osf.io/m72sh]. Reporting followed the STROBE Statement, the GRoLTS-Checklist, and a reporting checklist for HealthMeasures. The code we used to prepare the data and conduct the analyses was checked by an independent researcher, who filed a codecheck report.^21,22^

**FIGURE 1:**
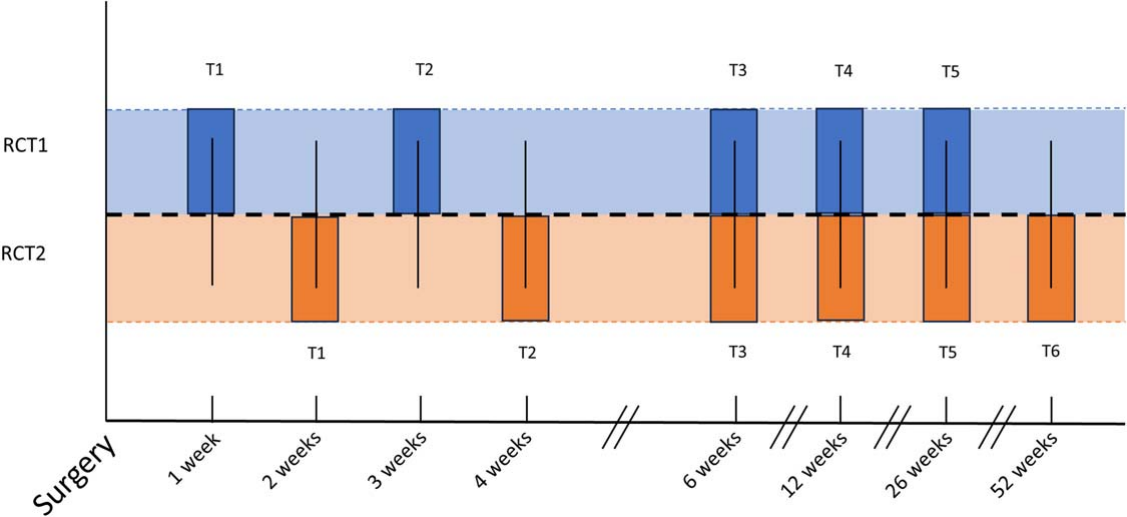
Overview of the different measurement times per trial in relation to each patient’s date of surgery.

The trials, referred to as RCT1^15^ and RCT2,^16^ aimed to evaluate the (cost-)effectiveness of ikHerstel in accelerating recovery after elective abdominal surgery. Recruitment ran from August 2015 to August 2016 for RCT1 and February 2016 to August 2017 for RCT2. Patients were identified from waiting lists of surgical and gynecological departments across 7-11 teaching hospitals in the Netherlands. Data were collected using electronic surveys administered at specific times relative to surgery. Measurement times differed between the trials. RCT2 included more invasive types of surgery and therefore, features an extra measurement time at 52 weeks. The measurement times T1 and T2 also lagged one week behind RCT1. Figure 1 illustrates these differences.

A total of 344 patients (RCT1) and 355 patients (RCT2) were included. Table 1 provides a detailed comparison of the inclusion and exclusion criteria for both trials.

**TABLE 1:** Inclusion and Exclusion Criteria Applied per Trial

Inclusion criteria		Exclusion criteria	
RCT1	RCT2	RCT1	RCT2
Aged 18–75 y	Aged 18–75 y	Surgery without a curative intention or with additional radiotherapy or chemotherapy	Surgery without a curative intention
On the waiting list for laparoscopic cholecystectomy	On the waiting list for open colectomy	Deep infiltrating endometriosis	Deep infiltrating endometriosis
On the waiting list for open inguinal hernia surgery	On the waiting list for laparoscopic colectomy	Ectopic pregnancy	Rectal surgery
On the waiting list for laparoscopic inguinal hernia surgery	On the waiting list for open hysterectomy	Adnexal surgery because of pelvic inflammatory disease or tubal ovarian abscess	Hysterectomy due to the presence of a malignancy
On the waiting list for laparoscopic adnexal surgery	On the waiting list for laparoscopic hysterectomy	Combination of several surgical procedures	Neoadjuvant therapy
		Severe comorbidity which might complicate the postoperative course	Combination of several surgical procedures
		Patients who are unable to understand the information from the study	Unable to use the Internet
		Insufficient understanding or ability to complete questionnaires in Dutch	Insufficient understanding or ability to complete questionnaires in Dutch

#### Variables

The primary outcome was physical functioning, operationalized through the Dutch-Flemish Patient-Reported Outcomes Measurement Information System-Physical Function (PROMIS-PF) item bank for adults (version 1.2).^23–26^ Specifically, we used a short form tailored to the needs of patients undergoing abdominal day surgery, which condenses the 121-item PROMIS-PF item bank into 29 activity items. Patients were able to customize the focus of their recovery by selecting 8 activities out of the 29. A frequency plot listing the activities selected by patients in the order of their frequency is provided in Supplemental Digital Content 1, http://links.lww.com/SLA/F415. Construct validity and responsiveness of this approach were confirmed in a previous study.^27^ We scored the short form using response-pattern scoring through the HealthMeasures Scoring Service with default calibrations. Lastly, we calculated Cronbach alpha at the baseline measure to evaluate the short form’s internal consistency in our patient populations.

Baseline patient characteristics included age (years), sex (male/female), level of education (low/medium/high), smoking behavior (yes/no), work type (paid work/no paid work), working hours (hrs/week), work satisfaction (bad/mediocre/good), type of surgery (laparoscopic adnexectomy/laparoscopic hernia inguinalis/open hernia inguinalis/cholecystectomy/total laparoscopic hysterectomy/abdominal hysterectomy/laparoscopic colectomy/open colectomy), complications (yes/no), perceived health (single item, self-rated score between 0 and 100), expectations for a full return to work (single item, self-rated number of expected days until full return to work), expectations for full recovery of normal activities (single item, self-rated number of expected days until recovery of all 8 activities), and difficulty of the selected PROMIS-PF activities (mean first threshold of the IRT item characteristic curve of the eight selected items on the baseline).

### Statistical Analyses

Statistical analyses were performed using R version 4.2.1. Data from the 2 trials were harmonized before analysis. Data analysis consisted of 2 steps: (1) longitudinal cluster analysis of PROMIS-PF t-scores to identify recovery patterns and (2) descriptive analysis to characterize clusters based on patient characteristics. Cluster analysis was performed using k-medoids clustering and growth mixture modeling (GMM).

#### Data Harmonization and Treatment of Missing Values

Harmonization was performed to safeguard data compatibility and content equivalence for each trial.^28^ Three researchers reviewed the results, and inconsistencies were addressed with the principal author. Missing data in the outcome were relatively low (RCT1: 13.2%, RCT2: 7.9%). We decided not to implement multiple imputations as its application in cluster analysis, where the goal is to assign n individuals into *k* homogenous groups rather than to estimate a population parameter, is a difficult proposition.^29^ Instead, cases with ≥3 missing PROMIS-PF t-scores in RCT1 or ≥4 in RCT2 were excluded, as they provided fewer than 3 data points.

#### Cluster Analysis

K-medoids clustering represents a variant of the popular k-means clustering analysis. Like k-means, k-medoids clustering is a data-adaptive (ie, nonparametric) hill-climbing algorithm which uses a dissimilarity measure to assign n observations to *k* clusters.^30,31^ K-medoids use of a real data point (the median) as its centroid recommended it to us, as this increases its interpretability, reduces its vulnerability to outliers, and reduces noise compared to GMM.^32^

We ran the k-medoids algorithm on longitudinal PROMIS-PF t-scores alone, using Euclidian distance across a 1 to 10 sequence for *k*. Each *k* clustering was iterated 20 times using random starting points for the median to avoid convergence to a local minimum. The optimal model was selected using the Bayesian Information Criterion, Akaike Information Criterion, and clinical relevance determined by visual inspection.^33,34^

Growth mixture modeling is a parametric approach to modeling longitudinal data. GMM approaches the assignment of individuals to trajectories on the basis of the conditional probability of that individual’s trajectory membership, which stems from an assumed distribution.^31,35,36^ Its estimation of a probability of cluster membership for each patient rather than k-medoids’ all-or-nothing cutoffs recommended it to us as a potentially more valid reflection of clinical reality.

We used Maximum Likelihood Estimation (MLE) with a grid search of 100 initial values to avoid convergence to local minima. Model selection was based on the Bayesian Information Criterion, the Akaike Information Criterion, and clinical relevance determined by visual inspection.^37,38^

#### Descriptive Analysis

To characterize patients within each cluster, we conducted descriptive analyses of their baseline characteristics. We report the mean and SD for normally distributed variables, the median [interquartile range (IQR)] for skewed variables, and frequencies (%) for categorical variables. As a test of intercluster differences, we used one-way Analysis of Variance (ANOVA) for normally distributed variables, Kruskal-Wallis ANOVA for skewed variables, and χ^2^ test for categorical variables. We applied a statistical significance threshold of α=0.05.

## RESULTS

### Study Sample

**FIGURE 2:**
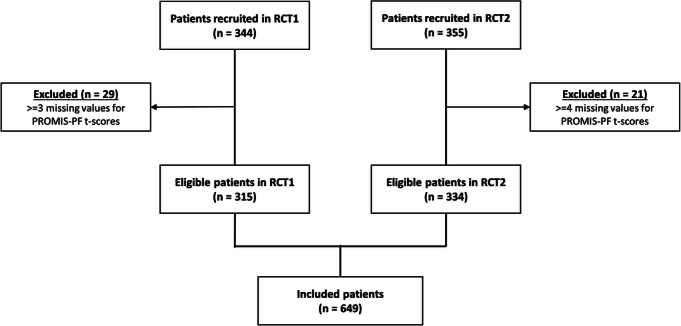
Patient flow diagram.

Figure 2 shows the patient flow diagram. The study included 649 patients: 315 from RCT1 and 334 from RCT2, after exclusion for missing data. Characteristics of excluded patients are shown in Supplemental Digital Content 2, http://links.lww.com/SLA/F415.

Table 2 summarizes the baseline characteristics of the final sample, stratified by trial. The majority of patients were female (54.6 in RCT1 and 70.7% in RCT2). Compared to RCT1, surgery in RCT2 was more invasive, the rate of complications was higher (16.5% vs 2.5%), and patients had lower expectations for the duration of their recovery (45.2 vs. 19.1 d). In addition, recovery of physical function was slower in RCT2. Cronbach alpha values for PROMIS-PF were high across both trials (0.88 for RCT1, 0.89 for RCT2). There seems to be little difference between trials in the difficulty of the PROMIS-PF activities that patients selected as part of their recovery plan.

**TABLE 2:** Patient Characteristics Per Trial

	Trial	
Variable*	RCT1 (n=315)	RCT2 (n=334)
Physical function; median [IQR]		
Baseline	52.4 [45.5;59.8]	50.0 [44.2;59.5]
1 week follow-up	41.4 [36.0;45.7]	–
2 weeks follow-up	–	37.6 [32.5;43.1]
3 weeks follow-up	50.4 [43.9;58.6]	–
4 weeks follow-up	–	43.8 [38.6;48.7]
6 weeks follow-up	57.5 [49.6;58.8]	48.8 [43.0;58.1]
12 weeks follow-up	59.2 [52.7;60.8]	58.4 [48.2;60.4]
26 weeks follow-up	–	59.0 [49.0;60.6]
52 weeks follow-up	–	59.1 [51.3;60.7]
Sex; n (%)		
male	143 (45.4%)	98 (29.3%)
female	172 (54.6%)	236 (70.7%)
Age; mean (sd)	50.3 (12.8)	52.7 (10.4)
Operation type; n (%)		
Adnex surgery	93 (29.5%)	–
Hernia inguinalis (laparoscopic)	121 (38.4%)	–
Hernia inguinalis (open)	3 (1.0%)	–
Cholecystectomy	98 (31.1%)	–
Hysterectomy (laparoscopic)	–	152 (45.5%)
Hysterectomy (open)	–	42 (12.6%)
Colectomy (laparoscopic)	–	117 (35.0%)
Colectomy (open)	–	23 (6.9%)
Smoking behaviour; n (%)		
Yes	57 (18.1%)	46 (13.8%)
No	258 (81.9%)	288 (86.2%)
Educational level; n (%)		
Low	30 (9.5%)	32 (9.6%)
Medium	125 (39.7%)	169 (50.6%)
High	160 (50.8%)	133 (39.8%)
Employment type; n (%)		
Paid work	234 (74.3%)	231 (69.2%)
No paid work	81 (25.7%)	103 (30.8%)
Working hours/week; median [IQR]	36.0 [26.0;40.0]	32.0 [26.0;40.0]
Work appraisal; n (%)		
Good	196 (82.7%)	197 (83.1%)
Fair	37 (15.6%)	36 (15.2%)
Mediocre	3 (1.3%)	2 (0.8%)
Bad	1 (0.4%)	2 (0.8%)
Expectations for full return to work; median [IQR]Median [IQR]	12.0 [7.0;14.0]	42.0 [28.0;42.0]
Expectations for full recovery of normal activities; median [IQR]Median [IQR]	14.0 [10.0;28.0]	42.0 [28.0;56.0]
Perceived health; median [IQR]	80.0 [65.0;90.0]	80.0 [60.0;90.0]
Adjuvant chemotherapy; n (%)		
No	–	300 (92.0%)
Yes	–	26 (8.0%)
NA	–	8
Complications; n (%)		
No	307 (97.5%)	278 (83.5%)
Yes	8 (2.5%)	55 (16.5%)
NA	0	1
Difficulty of the selected PROMIS-PF items; mean (sd)	−0.61 (0.06)	−0.60 (0.05)

* Data are presented as the mean and standard deviation for normally distributed variables, the median (interquartile range [IQR]) for skewed variables and frequencies (%) for categorical variables.

### Cluster Analysis

Cluster analysis extracted 3 to 6 clusters. Table 3 presents an overview of cluster labels and their similarities across trials and statistical techniques. Raw cluster plots are presented in Supplemental Digital Content 3 and 4, http://links.lww.com/SLA/F415. The iterative fit indices are presented in Supplemental Digital Content 5, http://links.lww.com/SLA/F415. Clusters A, B, and C appeared consistently across trials or statistical techniques. Cluster D emerged only in RCT2 while clusters E and F were found exclusively via k-medoids regardless of the trial. Alluvial plots (see Supplemental Digital Content 6, http://links.lww.com/SLA/F415) demonstrate the relative stability of k-medoids clusters compared to GMM clusters. These figures illustrate the assignment of patients to clusters across successive iterations of models. For example, the step-wise form of k-medoids’ cluster A in Supplemental Digital Content 6A, http://links.lww.com/SLA/F415 highlights its stability, as it is an illustration of the same patients remaining assigned to this cluster even as additional clusters emerge in subsequent models. In contrast, GMM’s cluster A, shown in Supplemental Digital Content 6B, http://links.lww.com/SLA/F415, demonstrates greater fluctuations: it emerges in the 3-cluster model, disappears in the 4-cluster model where its patients are reassigned to cluster B, and appears again in the 5-cluster model.

**TABLE 3:** Clustering Results and Similarities

Cluster	Label	K-medoids	GMM	RCT1	RCT2
A	Fast recovery	✔	✔	✔	✔
B	Intermediate recovery	✔	✔	✔	✔
C	Uneven recovery	✔	✔	✔	✔
D	Relapse	✔	✔	—	✔
E	Low gains	✔	—	✔	✔
F	High gains	✔	—	✔	✔

#### K-medoids Clustering

**FIGURE 3:**
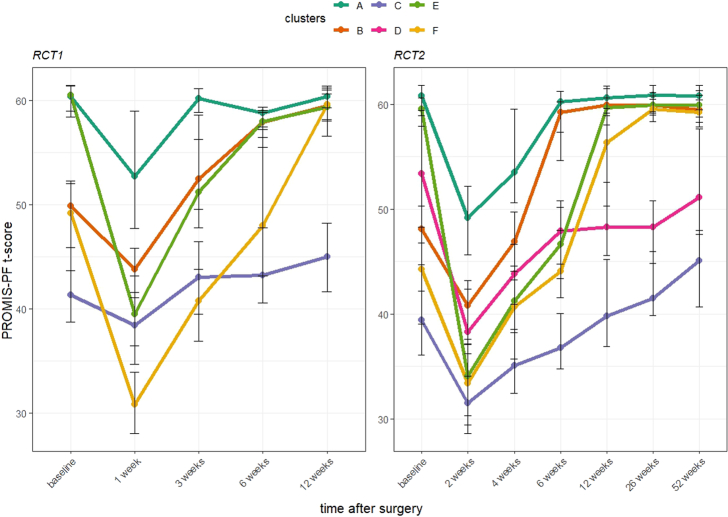
Longitudinal k-medoids clusters of PROMIS-PF t-scores over time.

K-medoids clustering identified five clusters in RCT1 and 6 in RCT2; these are illustrated in Figure 3. Patient characteristics of each cluster are detailed in Supplemental Digital Content 7, http://links.lww.com/SLA/F415. Clusters A, B, and C show similar physical functioning trajectories across trials. Clusters D through F varied between trials.

#### Cluster Discrimination

The clusters had similar sizes across RCT1 and RCT2, with significant differences in patient characteristics, including sex, age, expected return to work and perceived health. In RCT1, clusters additionally differed in level of education and expected return to normal activity, while in RCT2, differences were found in smoking behavior, employment type, and complication rates.

#### Cluster A: Fast Recovery

Cluster A is characterized by a high baseline score (median: 60.4, IQR: 3 for RCT1; median: 60.8, IQR: 2.9 for RCT2) followed by an intermediate decline (median change: −7.7 for RCT1; −11.6 for RCT2) and a sharp increase to T2 for RCT1 and T3 for RCT2. Patients plateaued after reaching their baseline score.

Cluster A comprised 18.0% of patients and in the case of RCT1, had the highest proportion of highly educated patients (79.3%). Return-to-work expectations were highest (7 d for RCT1; 28 d for RCT2), as were expectations for resuming normal activities and perceived health. RCT2’s cluster A additionally had the largest proportion of male patients (60.0%), laparoscopic colectomy surgeries (61.5%), and an older mean age (57.2 y).

#### Cluster B: Intermediate Recovery

Cluster B is characterized by an intermediate baseline score (median: 49.9, IQR: 6.4 for RCT1; median: 48.1, IQR: 5.6 for RCT2) followed by a slight decline (median change: −6.1 for RCT1; −7.3 for RCT2) and an intermediate to fast subsequent increase toward T2 (median: 52.5, IQR: 9 for RCT1; median: 46.9, IQR: 5.1 for RCT2). By T2 (RCT1) and T3 (RCT2), patients plateaued above the initial baseline score.

Cluster B comprised 26.3% of RCT1 patients and 18.9% of RCT2 patients, with intermediate age, intermediate recovery expectations, and intermediate perceived health. RCT2’s cluster B was comprised of mostly female patients (73.5%) and a concurrent overrepresentation of laparoscopic hysterectomies (51.0%).

#### Cluster C: Uneven Recovery

Cluster C is characterized by a low baseline score (median: 41.3, IQR: 8.2 for RCT1; median: 39.4, IQR: 6.1 for RCT2) with high variance and intermittent relapses. Baseline scores were followed by a slight decline (median change: −2.9 for RCT1; −7.9 for RCT2) and a gradual increase toward T2 for RCT1 and T4 for RCT2. Patients continue improving up to the final measurement times (median change: +3.7 for RCT1; +5.7 for RCT2).

Cluster C comprised 21.0% of RCT1 patients and 18.6% of RCT2 patients, with smokers marginally overrepresented. Patients had intermediate age and intermediate recovery expectations but the lowest perceived health (median: 66.5, IQR: 18.7 for RCT1; median: 60.0, IQR: 25 for RCT2). RCT1’s cluster C comprises 46.7% of all patients with a low educational level, while RCT2’s includes the largest proportion of patients experiencing complications.

#### Cluster D: Relapse (RCT2)

Cluster D describes a high baseline score (median: 53.4, IQR: 11.2) and a steep subsequent decline (median change: −15.1). It is characterized by the unevenness of recovery and the relapse exhibited by some patients T4 and T6 (outlier median change: −32.5). This relapse is not demonstrated by the median curve but is highlighted in the raw plots (Supplemental Digital Content 4D, http://links.lww.com/SLA/F415). By T6, most patients returned to baseline score but variance remains high.

Cluster D comprised 14.2% of RCT2 patients, with older age (mean: 57.2 y), and a significant proportion of colectomy surgeries. More than two-thirds of all adjuvant chemotherapy cases (69.2%) were assigned to this cluster. It holds the second-largest proportion of complications (27.4%) and the largest proportion of patients with no paid work (44.4%).

#### Cluster E: Low Gains

Cluster E bifurcated from cluster B in its k-1 cluster model (Supplemental Digital Content 6A and 6C, http://links.lww.com/SLA/F415). It shows many similarities with cluster B but differs in its baseline score, which was higher (median: 60.5, IQR: 3 for RCT1; median: 59.5 IQR: 2.7 for RCT2), and its decline, which was steeper (median change: −21 for RCT1; -25.5 for RCT2). Furthermore, patients in this cluster regained their baseline score later than patients in cluster B, at T3 for RCT1 and T4 for RCT2, where they plateaued.

Cluster E comprised 16.5% of RCT1 patients and 13.1% of RCT2 patients, of a young age, with slightly higher than average recovery expectations and intermediate to high perceived health.

#### Cluster F: High Gains

Cluster F is similarly bifurcated from cluster B. It describes a low baseline score (median: 49.2, IQR: 8.3 for RCT1; median: 44.2, IQR: 7.7 for RCT2) and a steep decline (median change: −18.4 for RCT1; −10.8 for RCT2) followed by a gradual increase across T2 and T3 to above baseline scores at T4 (median: 59.6, IQR: 2.7 for RCT1; median: 56.3, IQR: 9.3 for RCT2). In RCT2, patients plateaued at T5 and the IQR shrank.

Cluster F comprised 17.8% of RCT1 patients and 14.0% of RCT2 patients, of a slightly younger age compared to other clusters and with lower perceived health. In RCT2, the group is almost entirely comprised of female patients (96.9%), with a concurrently large share of gynecological surgeries. In RCT1, recovery expectations were lower (median: 21, IQR: 14) compared with other clusters.

#### Growth Mixture Modeling

**FIGURE 4:**
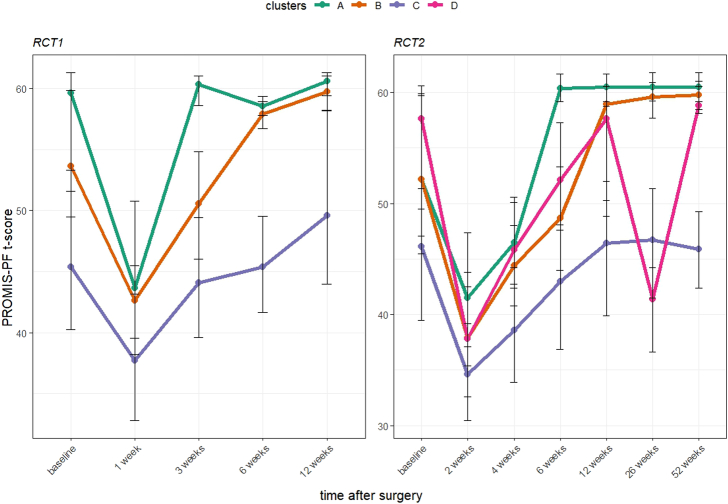
Growth mixture modeling clusters of PROMIS-PF t-scores over time.

Growth mixture modeling identified 3 clusters for RCT1 and 4 for RCT2, these are illustrated in Figure 4. Patient characteristics are detailed in Supplemental Digital Content 8, http://links.lww.com/SLA/F415. Clusters A, B, and C describe similar recovery trajectories across both trials, while cluster D was only observed in RCT2.

#### Cluster Discrimination

Cluster sizes vary significantly, with most patients found in cluster B. Differences between clusters across trials were only significant in terms of patients’ perceived health. In RCT1, clusters differed by education level, while in RCT2, there were additional differences in patients’ work type work type, expected return to work, expected return to normal activity, and adjuvant chemotherapy.

#### Cluster A: Fast Recovery

Cluster A is characterized by a high baseline score (median: 59.6 IQR: 8.0 for RCT1; median: 52.2, IQR: 13.5 for RCT2), followed by a steep decline (median change: −15.9 for RCT1; −10.1 for RCT2) and a steep increase to T2 (median: 60.3, IQR: 16.6 for RCT1) or T3 (median: 59.0, IQR: 8.5 for RCT2). Patients plateaued after reaching their baseline score.

Cluster A comprised 20.6% of RCT1 patients and 15.9% of RCT2 patients. There is a slightly disproportionate share of highly educated patients across trials. In RCT1, patients report slightly higher levels of perceived health, while in RCT2, patients report slightly higher expectations for recovery.

#### Cluster B: Intermediate Recovery

Cluster B is characterized by an intermediate to high baseline score (median: 53.7 IQR: 10.3 for RCT1; median: 52.2 IQR: 14.4 for RCT2), followed by an intermediate decline (median change: −11.1 for RCT1; −14.3 for RCT2) and a gradual increase, ending above baseline by T3 (median 57.9, IQR: 2.2 for RCT1) or T4 (median: 59.0, IQR: 8.5 for RCT2).

Cluster B comprised 44.4% of RCT1 patients and 52.4% of RCT2 patients. No distinct asymmetric distribution of patient characteristics across both trials was identifiable. For RCT2, perceived health is slightly lower (70.0).

#### Cluster C: Uneven Recovery

Cluster C is characterized by a low baseline score (median: 45.4 IQR: 11.4 in RCT1; median: 46.1 IQR: 11.8 in RCT2), followed by a decline (median change: −7.7 for RCT1; −11.5 for RCT2) and a gradual but uneven recovery. In RCT1, patients improve on their baseline score (med: 49.6 IQR: 14.1) while recovery in RCT2 ends at score similar to the baseline (med: 45.9 IQR: 6.9). Variance within this cluster is high, as evidenced from a consistently large IQR across measurement times.

Cluster C comprised 34.9% of RCT1 patients and 27.5% of RCT2 patients. In RCT1, a disproportionate share of low-education patients was found, and slightly lower perceived health. In RCT2, cluster C patients with no paid work are overrepresented (43.5%), and it contains close to two-thirds of all patients with adjuvant chemotherapy (61.5%) and close to half of all complications (41.8%).

#### Cluster D: Relapse (RCT2)

Cluster D is characterized by a high baseline score (median: 57.6, IQR: 10.2), followed by a steep decline (median change: −19.8) and uneven progression of physical function to T4 before a second decline occurs to T5 (med change: −16.2) followed by a complete recovery at T6 (med: 58.5, IQR: 2.4). Variance is high throughout the cluster except at its end.

Cluster D comprised only 14 patients (4.2%), with slightly low recovery expectations. Little over a quarter (28.6%) of this group underwent complications and 38.5% were treated with adjuvant chemotherapy.

## DISCUSSION

This study demonstrates the heterogeneity of functional recovery for patients following elective abdominal surgery, in the midst of which we identified distinct recovery patterns. Three patterns (A, B, and C) were consistently extracted, regardless of statistical techniques or the severity of surgery. These we would designate as fast, intermediate, and uneven recovery, or clusters A, B and C. The relapse pattern (cluster D) appeared consistently across methods but was specific to the severe surgeries of RCT2. Conversely, the low gains (cluster E) and high gains (cluster F) patterns were extracted regardless of surgical severity but only through k-medoids clustering. The low gains pattern describes a group of patients who in spite of their high degree of preoperative physical functioning, show a steep decline following surgery and a slow subsequent recovery. Conversely, the high-gain pattern describes a group of patients with low levels of preoperative functioning who recover equally slowly but to a point well above their baseline.

Emergent differences in patient characteristics provide further insight into these patterns. The relapse pattern’s high prevalence of chemotherapy and complications seems illustrative of its uneven course of recovery: high variance of physical function throughout and a sudden drop of function at 26 weeks after surgery. Likewise, the high levels of perceived health and elevated expectations found in the fast pattern are consistent with the perspective of psychosocial factors as positive determinants of recovery after surgery.^39,40^ The low preoperative perception of health in the uneven pattern meanwhile describes the other side of this medallion. Contrary to established knowledge, baseline physical function does not seem predictive of recovery for each pattern: the high-gain group recovers well despite low initial functioning, while the low-gain group shows delayed recovery despite their high physical function at baseline.^39^ This may be due to a ceiling effect, curbing growth in physical function for both clusters beyond a ceiling value and effectively allowing more room for improvement to high-gain patients compared with low-gain patients. This would also explain why the fast and intermediate groups plateau at this same level of physical function. It may be a characteristic of patients’ recovery plans, indicated by the relatively low difficulty score of the selected PROMIS-PF activities. P may have exhibited a predilection for easy activities when designing their personalized recovery plans, thereby leading to a ceiling effect in measuring physical function. The high frequency of low-intensity activities like personal care and the low frequency of high-intensity activities like running 8 km in Supplemental Digital Content 1, http://links.lww.com/SLA/F415 corroborate this hypothesis. No differences between patterns were found in terms of operation types, regardless of statistical technique. This is inconsistent with previous research on postoperative recovery following gynecological surgery, which showed an association between surgical invasiveness and return to work.^41^ It may be explained by our use of physical function rather than return to work as an outcome measure, as a more recent study using the Recovery Index likewise found no such relationship.^40^

Other studies have found distinct patterns of recovery following surgery. A longitudinal study on recovery trajectories for a mixed surgical cohort found 3 pain trajectories, which included moderate, mild, and low impairment of physical function.^42^ Another, focused on recovery after hip surgery found 4 groups corresponding to very good, good, poor, and very poor functioning.^43^ Yet another investigating recovery after knee arthroplasty found 3.^44^ A study on veterans in the United States of America likewise found 5 patterns, using as its outcome the days patients spend outside of their home due to their surgery.^45^ The patient subgroups inferred by those patterns, however, seem incomparable to our own due to the wide differences between our outcome measures and study samples.

Our approach and results build on these studies in a number of ways. To our knowledge, no previous study has modeled physical functioning after abdominal surgery. Combining data representing different severities of abdominal surgery furthermore provides a broad overview of the effects of surgery on physical functioning and advocates for the broad applicability of our results. It may explain the relatively large number of clusters we identified, clusters that go beyond the typical slow, intermediate, and fast patterns of recovery to capture patients with highly uneven or even relapsing patterns of recovery. Our inclusion of psychosocial variables like patients’ expectations for recovery also adds weight to our findings, as these emerged as consistent descriptions of intercluster differences. Lastly, our utilization of 2 clustering techniques and 2 trials contextualizes each cluster we find. The consistent extraction of certain clusters, regardless of either surgical invasiveness or statistical approach, adds to their validity.

Several limitations must be addressed. Bias may have been introduced by our removal of patients with ≥3 or ≥4 missing values for PROMIS-PF. Comparison of removed versus included patients (Supplemental Digital Content 2, http://links.lww.com/SLA/F415) shows few differences between removed and included patients, but the significant test for complications is notable, as it indicates patients suffering from complications may have foregone reporting their recovery process. A second limitation arises from the disparity in measurement times between RCT1 and RCT2. This made concatenation of the trials ill-advised, thus necessitating stratified analyses. The resultant reduction in power may have increased the risk of type 2 error while testing for differences in patient characteristics between clusters. A third limitation pertains to the potential for subjectivity introduced in the process of model selection. This may explain the differences between our GMM and k-medoids clusters, rather than simply GMM’s proclivity to extract fewer clusters.^46^ For example, in trial RCT2, we chose a 4-cluster GMM model—despite the additional cluster D’s instability and small size—as it seemed to capture an important and unexpected feature of recovery within our research population: relapse. To increase the transparency and replicability of our research, we published our process and the data visualizations that guided it on Open Science Framework (https://osf.io/kd5x8).

### Future Implications

These patterns could serve as a roadmap to health care professionals, facilitating shared decision-making by visualizing the heterogeneity of postsurgical convalescence. Future researchers may use them as targets for prediction, leveraging big data to forecast recovery in a longitudinal space rather than a dichotomous endpoint. A multidimensional approach, incorporating aspects like pain, anxiety, or depression alongside physical function, would go even further to capture the full spectrum of recovery. In addition to benefiting shared decision-making, this data-driven approach to perioperative care may pave the way for patient selection, identifying patients who would benefit most and potentially excluding those unlikely to gain significant benefits from surgery.

## CONCLUSIONS

Distinct patterns of longitudinal recovery following abdominal surgery were identified, regardless of surgical invasiveness or clustering technique. We have typified these as fast, intermediate, and intermittent recovery. In the case of major surgery, an additional relapse cluster was found. Using machine learning clustering, we identified 2 additional clusters representing patients with slow recovery despite high preoperative physical functioning, and patients with recovery beyond their preoperative level of physical functioning. Taking stock of our limitations, future research may build on these results by validating these patterns on a larger data set, using computerized adaptive testing to minimize the potential for ceiling effects in measuring physical function, and employing a systematic approach to substantive interpretation of the results. Once their validity is established, predictive modeling may link these patterns to risk factors to enhance shared decision-making and patient selection in a way that reflects the longitudinal character of postoperative recovery.

## Supplementary Material
