# Supplementary material for: Recovery Patterns: Longitudinal Cluster Analysis of Physical Function Following Abdominal Surgery
Source: Ann Surg. 2025 Feb 18;284(1):51–60. doi: 10.1097/SLA.0000000000006671 (PMC13258088; doi:10.1097/SLA.0000000000006671)
Supplement: Supplementary file 1 [file sla-284-051-s001.docx]

## **
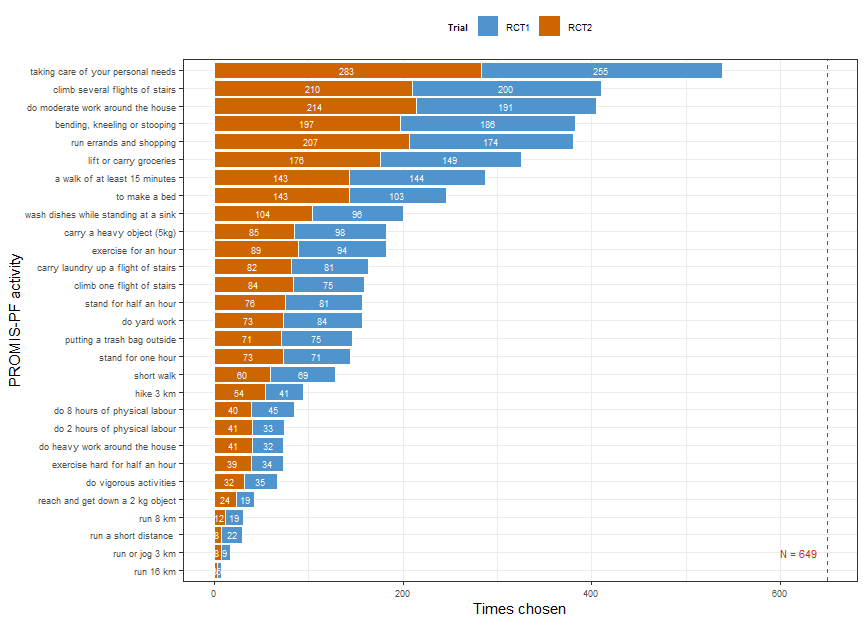
SDC 1:** Frequency plot of PROMIS-PF items selected by patients, stratified per trial

## **SDC 2:** Comparison of included patients versus patients removed due to missingness in PROMIS-PF outcome

| **Variable** | **Metric*** | **RCT1** | | | **RCT2** | | |
| --- | --- | --- | --- | --- | --- | --- | --- |
|  |  | Included (n = 315) | Removed  (n = 29) | p-value** | Included (n = 334) | Removed  (n = 21) | p-value** |
| Group | Control | 162 (51.4%) | 9 (31.0%) | .051 | 171 (51.2%) | 6 (28.6%) | .070 |
|  | Intervention | 153 (48.6%) | 20 (69.0%) |  | 163 (48.8%) | 15 (71.4%) |  |
| Sex | Male | 143 (45.4%) | 14 (48.2%) | .891 | 98 (29.3%) | 8 (38.1%) | .461 |
|  | Female | 172 (54.6%) | 15 (51.8%) |  | 236 (70.7%) | 13 (61.9%) |  |
| Age | Mean (sd) | 50.3 (12.8) | 52.6 (12.8) | .841 | 52.7 (10.4) | 58.6 (12.6) | .662 |
| Surgery type | Adnex surgery | 93 (29.5%) | 10 (34.5%) | .780 | - | - | - |
|  | Hernia inguinalis (laparoscopic) | 121 (38.4%) | 9 (31.0%) |  | - | - |  |
|  | Hernia inguinalis (open) | 3 (1.0%) | 0 |  | - | - |  |
|  | Cholecystectomy | 98 (31.1%) | 10 (34.5%) |  | - | - |  |
|  | Hysterectomy (laparoscopic) | - | - | - | 152 (45.5%) | 7 (33.3%) | .338 |
|  | Hysterectomy (open) | - | - |  | 42 (12.6%) | 3 (14.3%) |  |
|  | Colectomy (laparoscopic) | - | - |  | 117 (35.0%) | 11 (52.4%) |  |
|  | Colectomy (open) | - | - |  | 23 (6.9%) | 0 |  |
| Smoking behaviour | Yes | 57 (18.1%) | 7 (24.1%) | .454 | 46 (13.8%) | 2 (9.5%) | .752 |
|  | No | 258 (81.9%) | 22 (75.9%) |  | 288 (86.2%) | 19 (90.5%) |  |
| Education level | Low | 30 (9.5%) | 3 (10.3%) | .920 | 32 (9.6%) | 5 (23.8%) | .075 |
|  | Medium | 125 (39.7%) | 12 (41.4%) |  | 169 (50.6%) | 7 (33.3%) |  |
|  | High | 160 (50.8%) | 14 (48.3%) |  | 133 (39.8%) | 9 (42.9%) |  |
| Work type | Paid work | 234 (74.3%) | 18 (62.1%) | .187 | 231 (69.2%) | 6 (28.6%) | **<.001** |
|  | No paid work | 81 (25.7%) | 11 (37.9%) |  | 103 (30.8%) | 15 (71.4%) |  |
| Work hours / week | Median [IQR] | 36.0 [26.0;40.0] | 36.0 [31.0;40.0] | .420 | 32.0 [26.0;40.0] | 28.5 [24.2;35] | .548 |
| Work appraisal | Good | 196 (82.7%) | 15 (83.3%) | .367 | 197 (83.1%) | 4 (66.7%) | .316 |
|  | Fair | 37 (15.6%) | 2 (11.1%) |  | 36 (15.2%) | 2 (33.3%) |  |
|  | Mediocre | 3 (1.3%) | 1 (5.6%) |  | 2 (0.8%) | 0 |  |
|  | Bad | 1 (0.4%) | 0 |  | 2 (0.8%) | 0 |  |
| Expectations for full return to work (RTW) | Median [IQR] | 12.0 [7.0;14.0] | 14.0 [7.0;14.0] | .403 | 42.0 [28.0;42.0] | 42.0 [29.2;42.0] | .939 |
| Expectations for full recovery of normal activities (RNA) | Median [IQR] | 14.0 [10.0;28.0] | 14.0 [14.0;28.0] | .144 | 42.0 [28.0;56.0] | 42.0 [28.0;42.0] | .442 |
| Perceived health | Median [IQR] | 80.0 [65.0;90.0] | 80.0 [60.0;85.0] | .570 | 80.0 [60.0;90.0] | 75.0 [50.0;85.0] | .243 |
| Adjuvant Chemotherapy | No | - | - | - | 300 (92.0%) | 18 (85.7%) | .402 |
|  | Yes | - | - |  | 26 (8.0%) | 3 (14.3%) |  |
|  | NA | - | - |  | 8 | 0 |  |
| Complications | No | 307 (97.5%) | 27 (93.1%) | .203 | 278 (83.5%) | 11 (52.4%) | **.001** |
|  | Yes | 8 (2.5%) | 2 (6.9%) |  | 55 (16.5%) | 10 (47.6%) |  |
|  | NA | 0 | 0 |  | 1 | 0 |  |
| Difficulty of the selected PROMIS-PF items | Mean (sd) | -0.61 (0.06) | -0.61 (0.08) | .697 | -0.60 (0.05) | -0.62 (0.06) | .211 |

***** data are presented as the mean and standard deviation for normally distributed variables, the median (interquartile range [IQR]) for skewed variables and frequencies (%) for categorical variables.

**Student’s t-test for normally distributed variables, Mann-Whitney U-test for non-normally distributed variables, Fisher’s exact test for categorical variables.

## **SDC 3:** Raw cluster plots of RCT1

**Cluster A1:** *fast pattern – k-medoids*


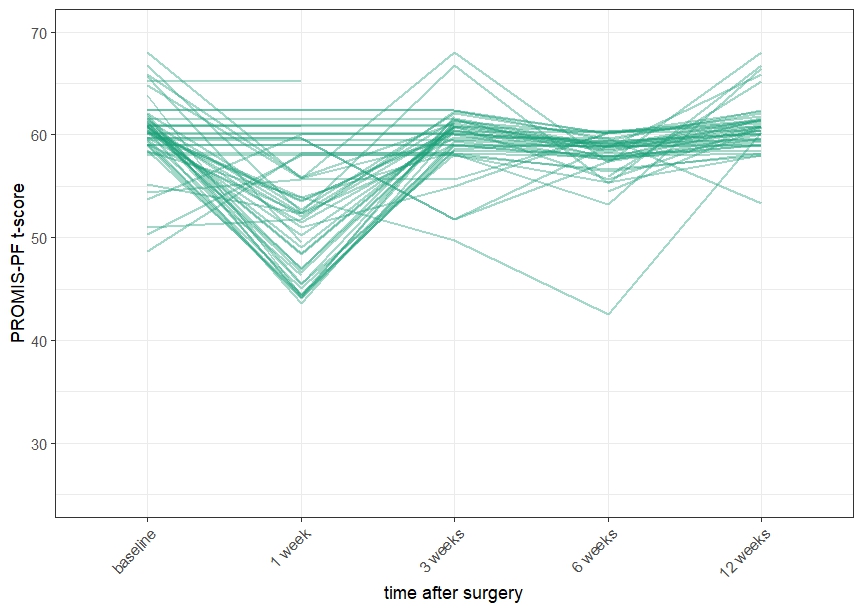


**Cluster A2:** *fast pattern - GMM*


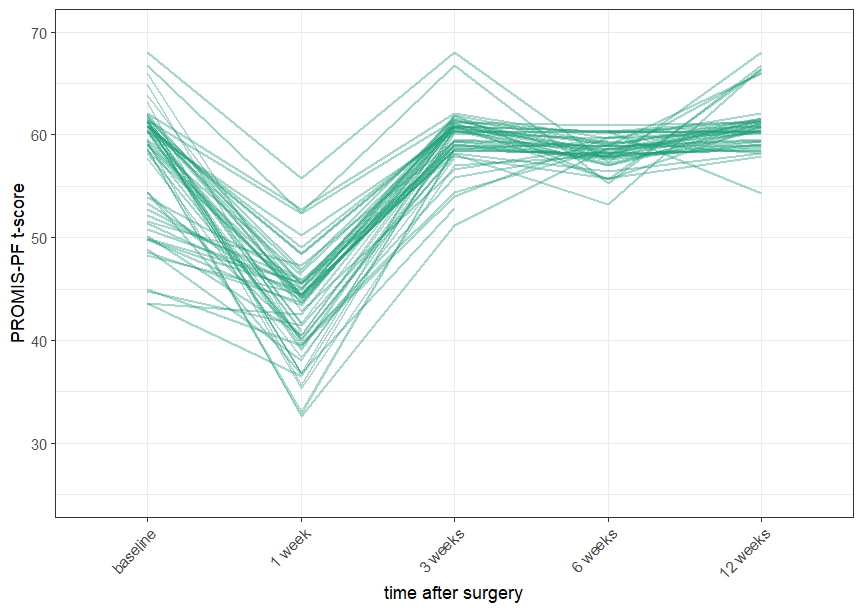


**Cluster B1:** *intermediate pattern – k-medoids*


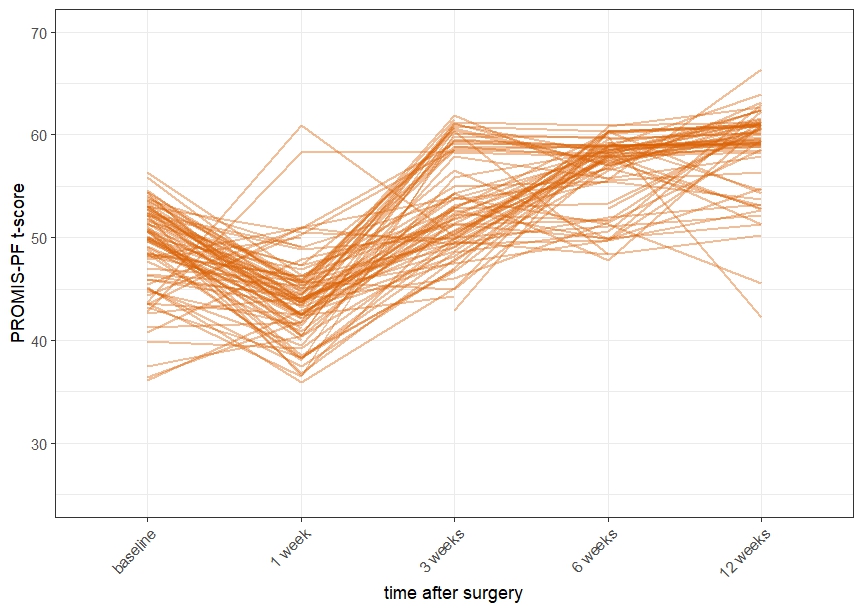


**Cluster B2:** *intermediate pattern – GMM*


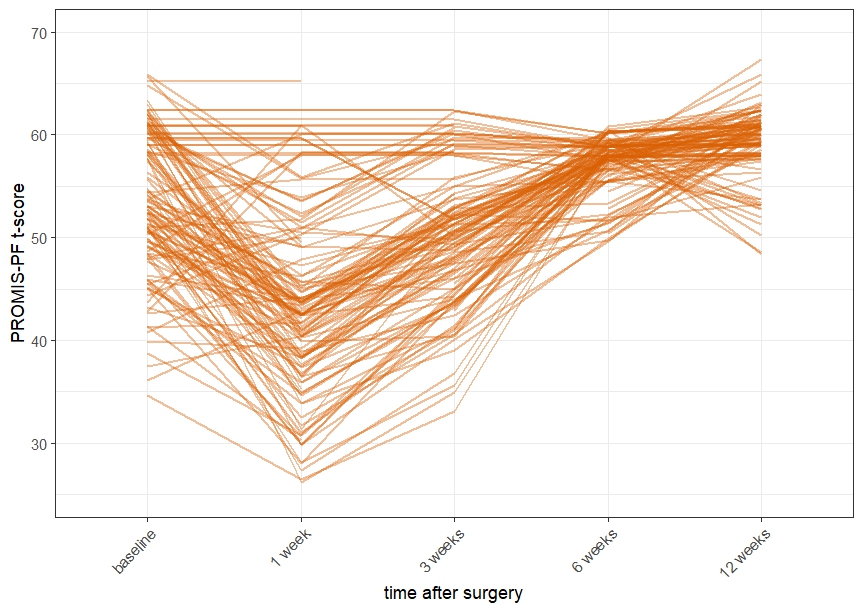


**Cluster C1:** *uneven pattern – k-medoids*


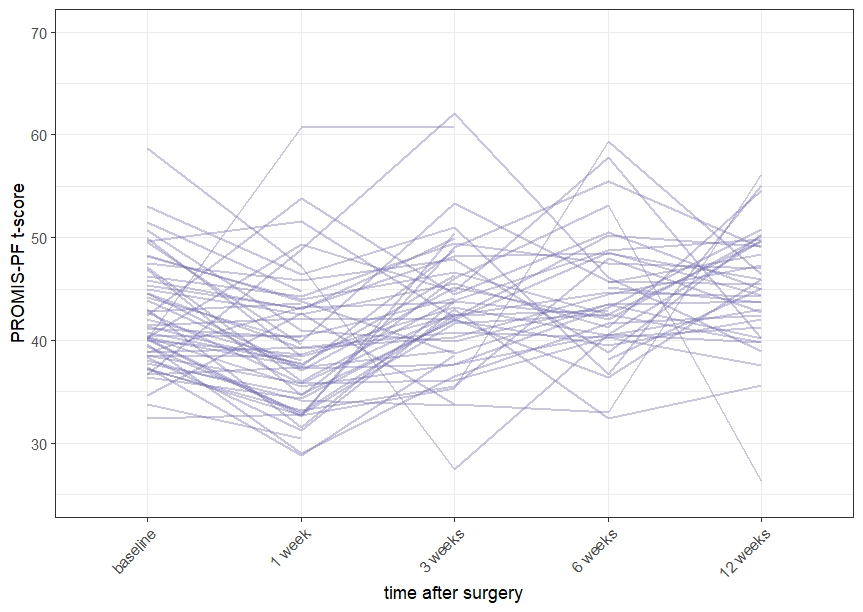


**Cluster C2:** *uneven pattern – GMM*


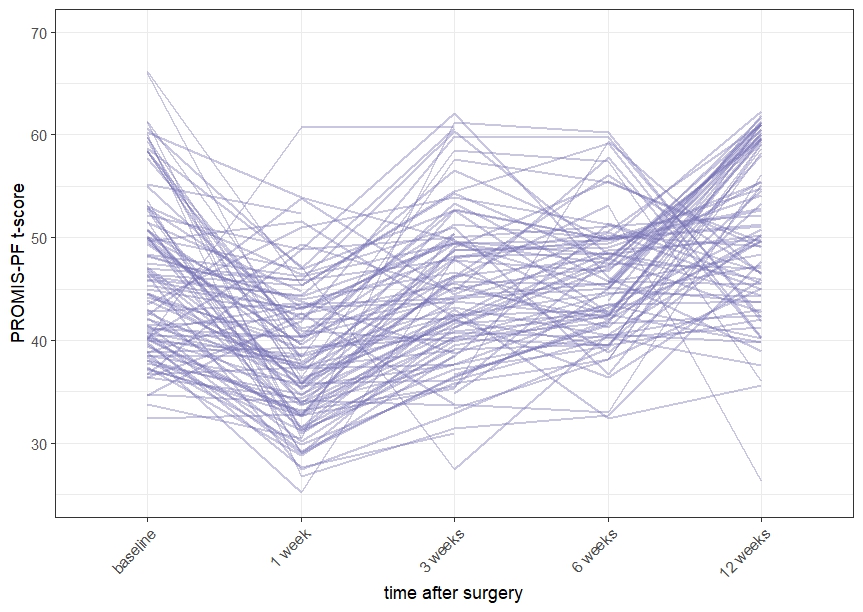


**Cluster E: *low gains – k-medoids***


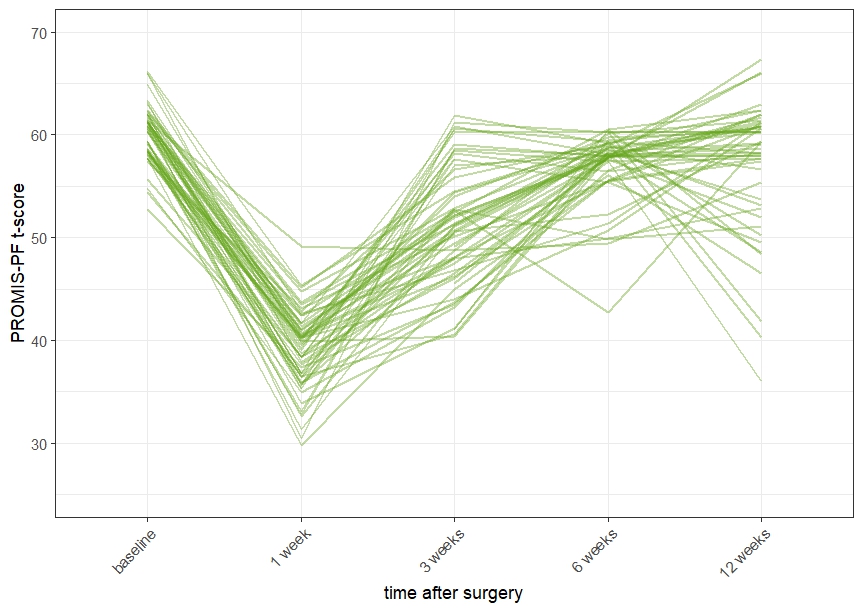


**Cluster F:** *high gains – k-medoids*


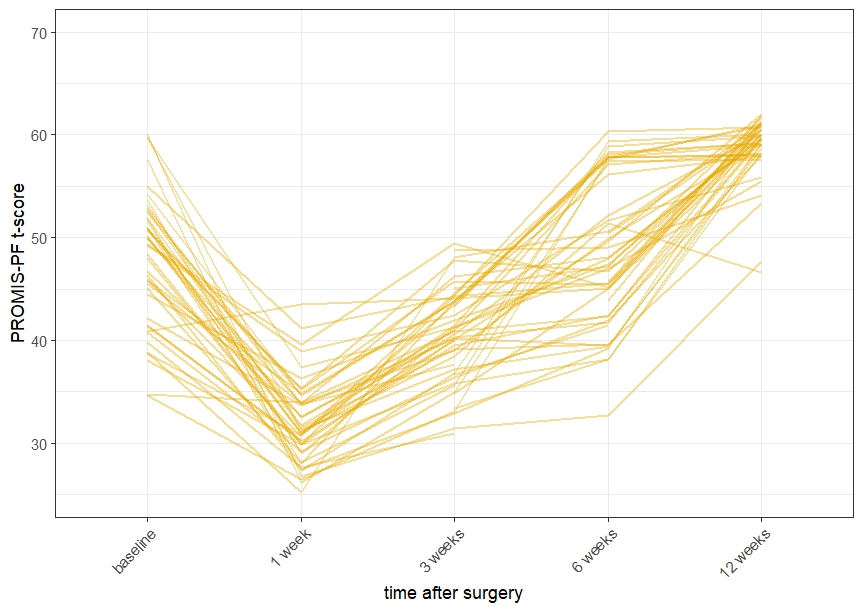


## **SDC 4:** Raw cluster plots of RCT2

**Cluster A1:** *fast pattern – k-medoids*
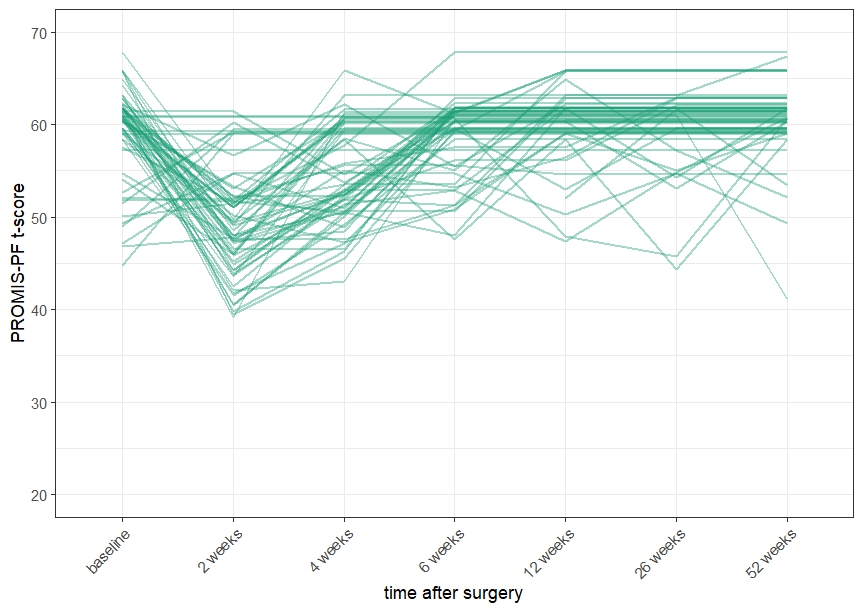


**Cluster A2:** *fast pattern - GMM*


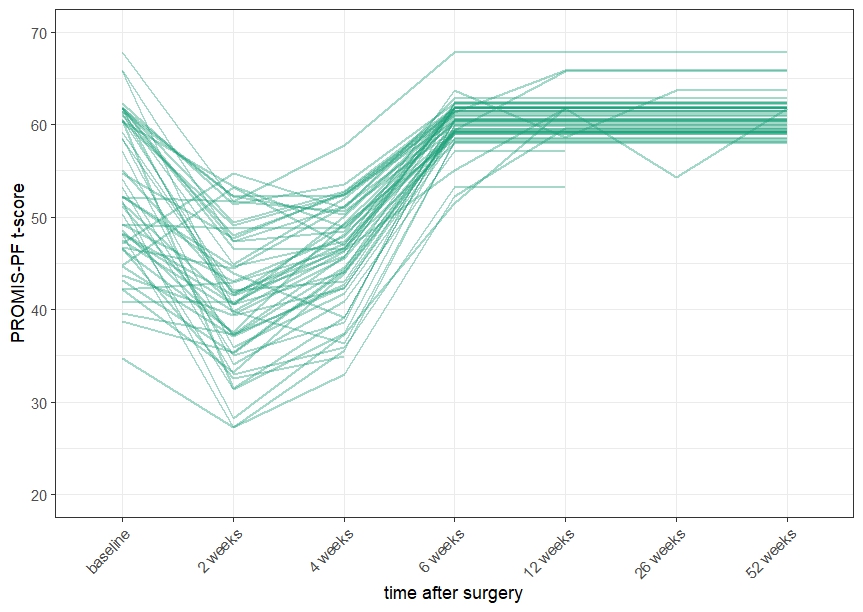


**Cluster B1:** *intermediate pattern – k-medoids*


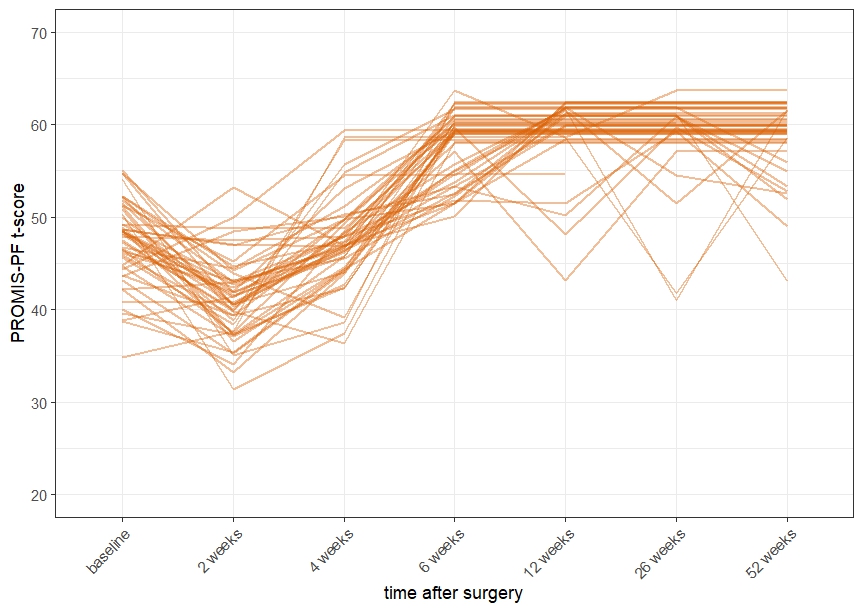


**Cluster B2:** intermediate pattern - GMM


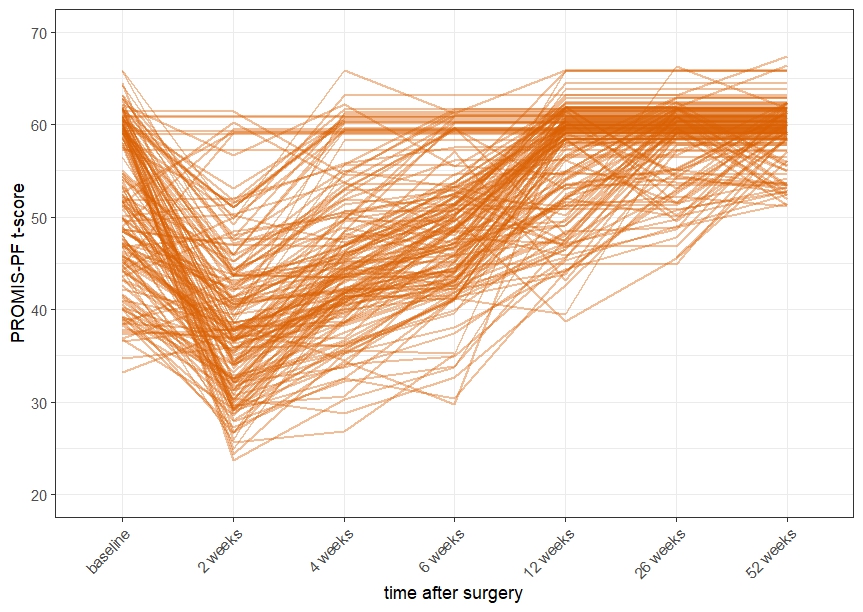


**Cluster C1:** *uneven pattern – k-medoids*


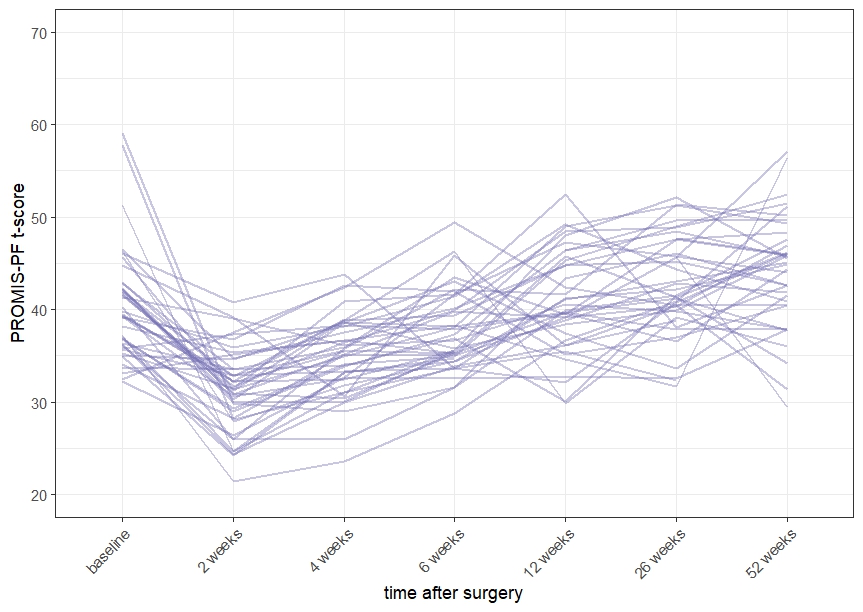


**Cluster C2:** *uneven pattern - GMM*


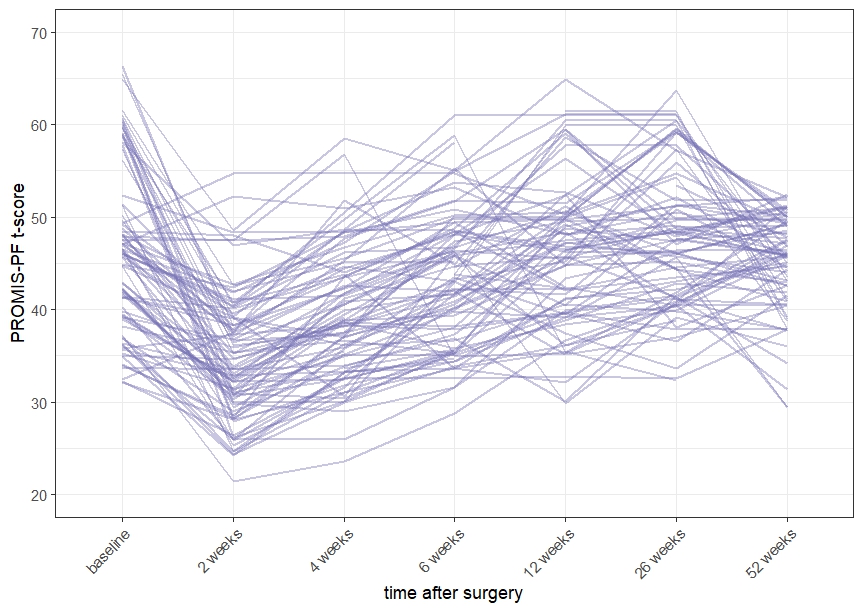


**Cluster D1:** *relapse pattern – k-medoids*


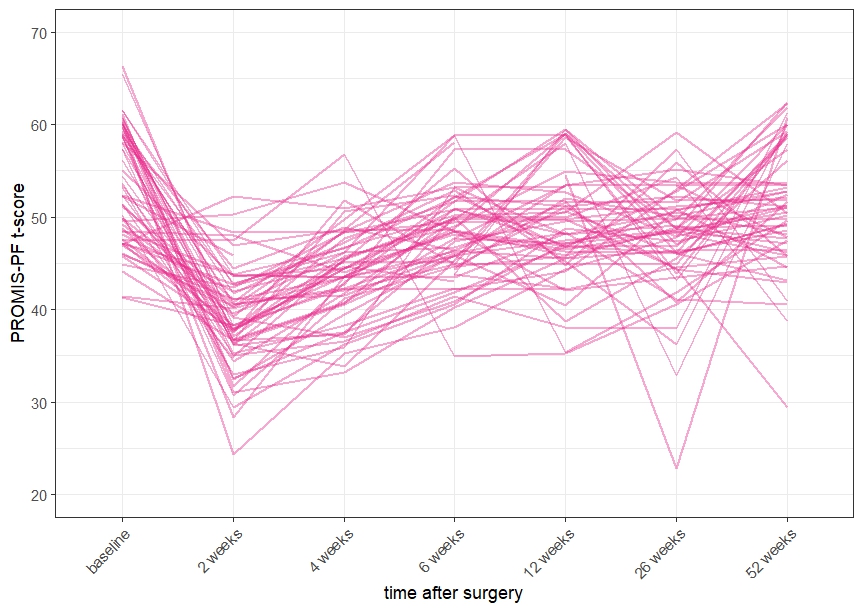


**Cluster D2:** *relapse pattern - GMM*


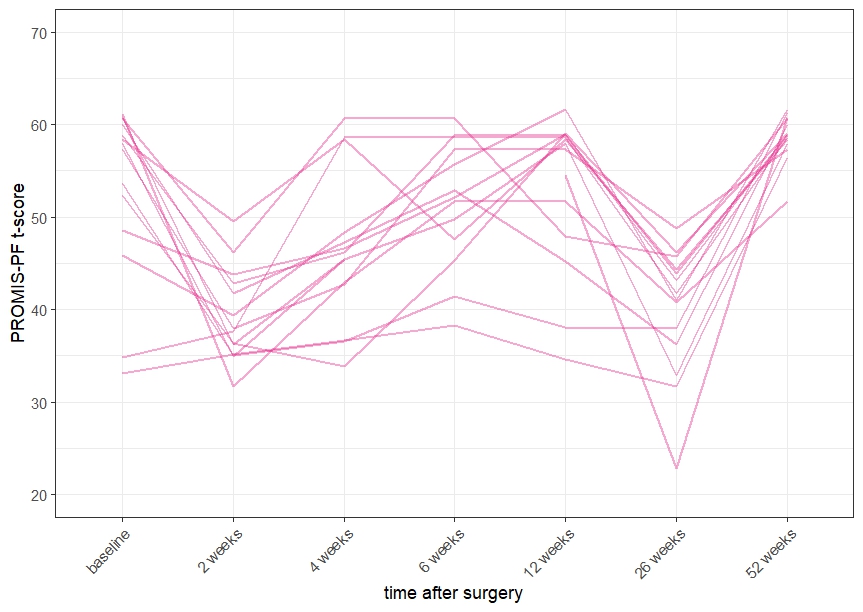


**Cluster E:** *low gains – k-medoids*


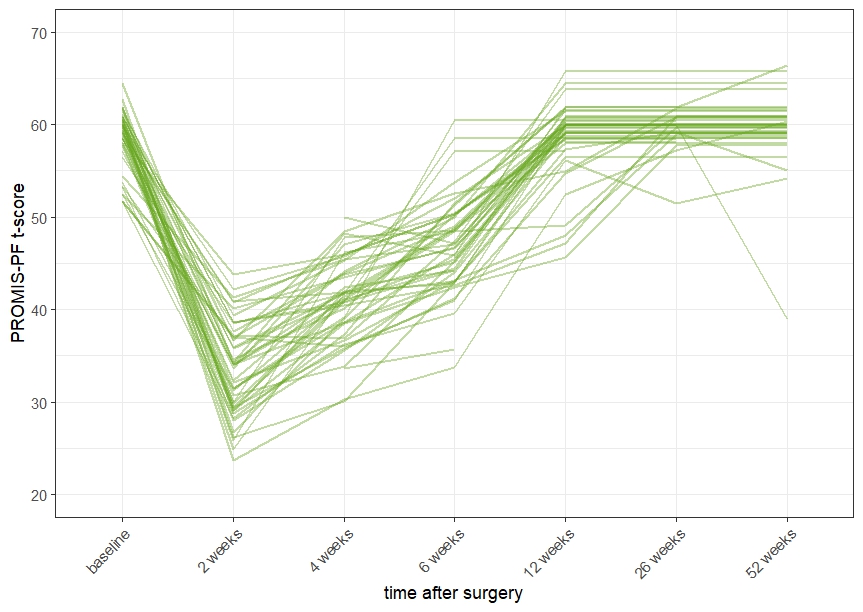


**Cluster F:** *high gains – k-medoids*
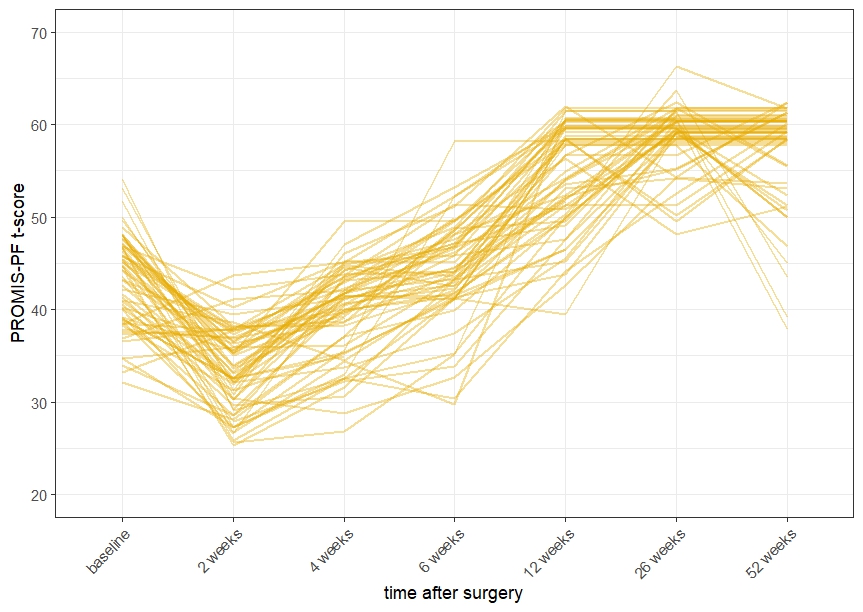


## **SDC 5:** BIC and AIC fit indices for k-medoids clustering & GMM

##
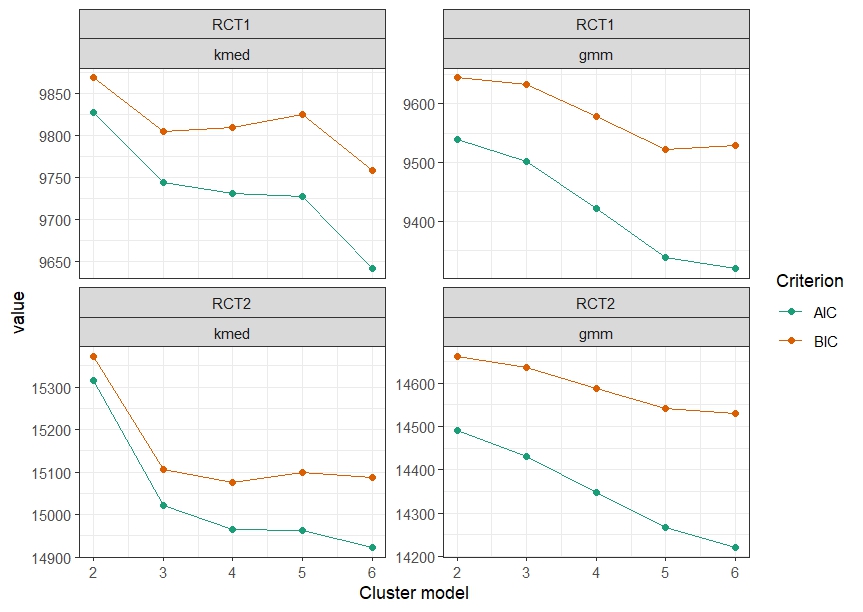


## **SDC 6:** Alluvial plots

A K-medoids clustering – RCT1


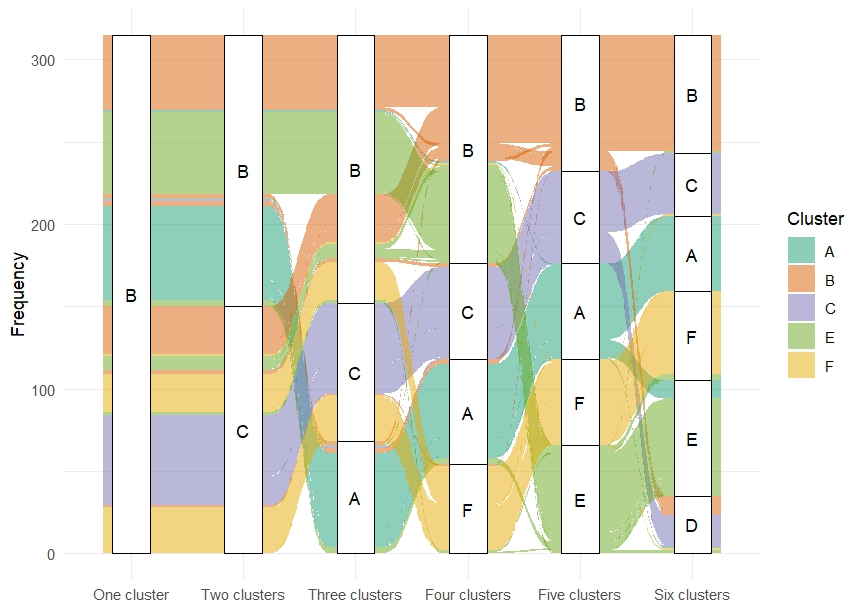


B GMM – RCT1


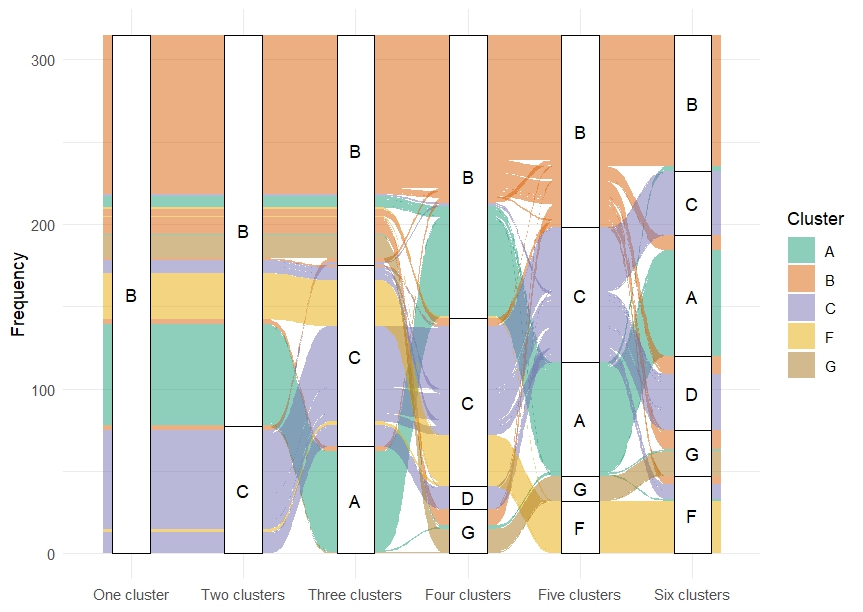


C K-medoids clustering – RCT2


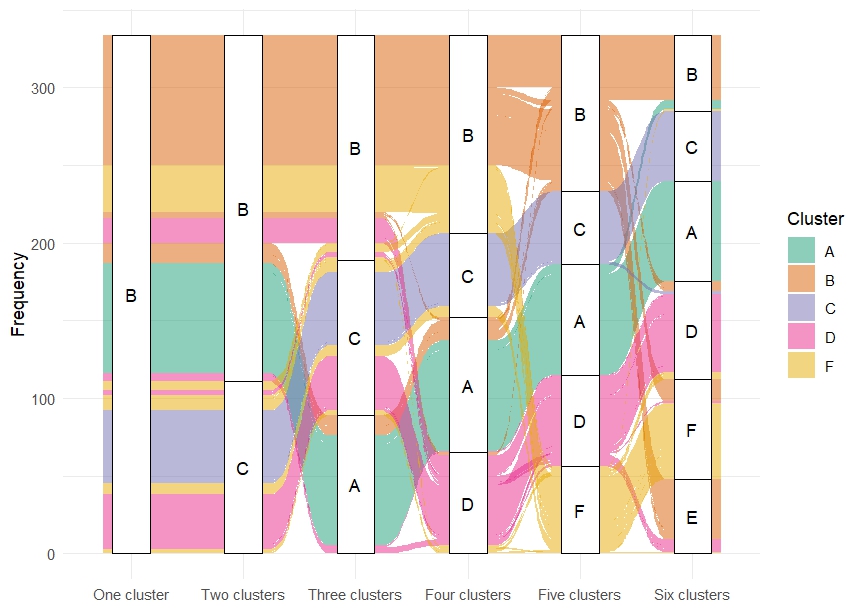


D GMM – RCT2


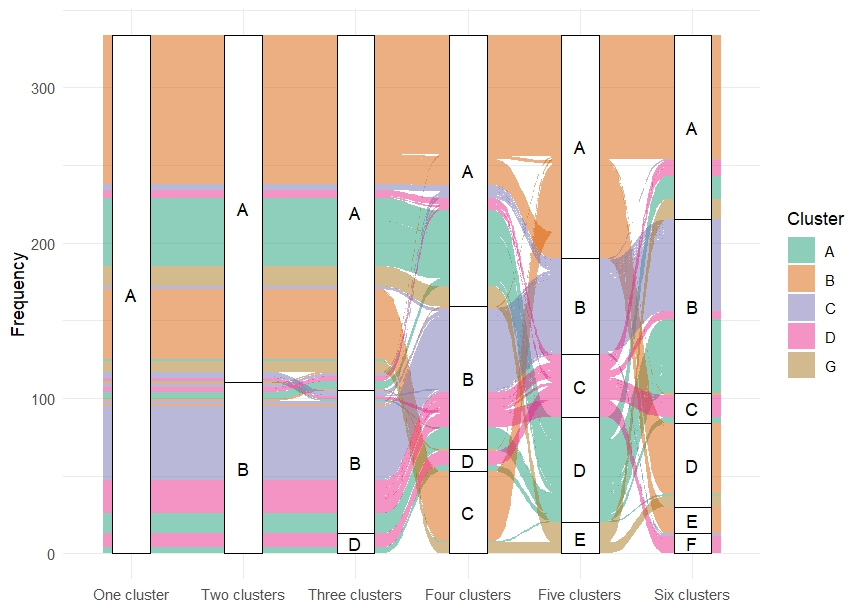


## **SDC 7:** k-medoids cluster characteristics

| Variable | Metric* | Clusters | | | | | | | | | | | | |
| --- | --- | --- | --- | --- | --- | --- | --- | --- | --- | --- | --- | --- | --- | --- |
|  |  | RCT1 | | | | | | RCT2 | | | | | | |
|  |  | A  *Fast*  n=58 (18.4%) | B  *Intermediate*  n=83 (26.3%) | C  *Uneven*  n=66 (21.0%) | E  *Low gains*  n=52 (16.5%) | F  *High gains*  n=56 (17.8%) | p ** | A  *Fast*  n=63 (18.3%) | B  *Intermediate*  n=65 (18.9%) | C  *Uneven*  n=64 (18.6%) | D  *Relapse*  n=49 (14.2%) | E  *Low gains*  n=45 (13.1%) | F  *High gains*  n=48 (14.0%) | p** |
| Physical function | Baseline | 60.4 [59.0;61.5] | 49.9 [45.9;52.3] | 41.3 [38.7;45.9] | 60.5 [58.4;61.4] | 49.2 [43.7;52.0] | **<.001** | 60.8 [58.9;61.8] | 48.1 [44.7;50.3] | 39.4 [36.1;42.2] | 53.4 [48.2;59.4] | 59.5 [57.9;60.6] | 44.2 [39.1;46.8] | **<.001** |
|  | 1 week | 52.7 [47.7;59.0] | 43.8 [41.6;45.8] | 38.4 [34.7;43.2] | 39.5 [36.5;41.0] | 30.8 [28.0;33.9] | **<.001** | - | - | - | - | - | - | **-** |
|  | 2 weeks | - | - | - | - | - | **-** | 49.2 [45.6;52.2] | 40.8 [37.6;43.2] | 31.5 [28.6;34.0] | 38.3 [36.2;42.4] | 34.0 [29.4;37.2] | 33.4 [30.3;37.1] | **<.001** |
|  | 3 weeks | 60.2 [58.9;61.1] | 52.5 [49.6;58.6] | 43.0 [39.5;46.5] | 51.2 [47.8;56.2] | 40.8 [36.9;43.8] | **<.001** | - | - | - | - | - | - | **-** |
|  | 4 weeks | - | - | - | - | - | **-** | 53.5 [50.6;59.5] | 46.9 [44.6;49.7] | 35.1 [32.5;38.2] | 43.8 [40.9;46.7] | 41.2 [38.5;44.1] | 40.7 [35.7;43.2] | **<.001** |
|  | 6 weeks | 58.8 [57.5;59.4] | 57.9 [55.5;58.9] | 43.2 [40.5;47.8] | 58.0 [56.5;59.0] | 48.0 [43.1;57.2] | **<.001** | 60.2 [57.4;61.2] | 59.2 [54.6;60.2] | 36.8 [34.8;40.1] | 47.9 [44.8;50.8] | 46.7 [43.4;50.2] | 44.1 [41.5;48.0] | **<.001** |
|  | 12 weeks | 60.4 [59.3;61.4] | 59.5 [58.2;61.0] | 45.0 [41.6;48.2] | 59.4 [56.6;61.2] | 59.6 [58.0;60.7] | **<.001** | 60.6 [59.2;61.7] | 59.9 [58.9;61.5] | 39.8 [36.9;45.2] | 48.2 [45.5;52.5] | 59.7 [58.0;60.6] | 56.3 [50.3;59.6] | **<.001** |
|  | 26 weeks | - | - | - | - | - | - | 60.8 [59.3;61.8] | 59.9 [59.2;61.1] | 41.5 [39.8;46.0] | 48.3 [44.8;50.8] | 59.9 [58.9;60.8] | 59.5 [58.3;60.5] | **<.001** |
|  | 52 weeks | - | - | - | - | - | - | 60.8 [59.5;61.8] | 59.5 [58.2;61.3] | 45.1 [40.6;48.0] | 51.1 [47.6;57.6] | 59.9 [58.8;60.8] | 59.2 [57.9;60.4] | **<.001** |
| Sex | Male | 30 (51.7%) | 52 (44.6%) | 23 (60.7%) | 24 (53.0%) | 14 (59.6%) | **<.001** | 39 (60.0%) | 13 (26.5%) | 7 (15.6%) | 30 (47.6%) | 7 (14.6%) | 2 (3.1%) | **<.001** |
|  | Female | 28 (48.3%) | 31 (55.4%) | 33 (39.3%) | 42 (47.0%) | 38 (40.4%) |  | 26 (40.0%) | 36 (73.5%) | 38 (84.4%) | 33 (52.4%) | 41 (85.4%) | 62 (96.9%) |  |
| Age | Mean (sd) | 50.9 (11.2) | 53.0 (13.0) | 51.6 (12.8) | 48.2 (13.0) | 46.2 (12.5) | **.019** | 57.2 (10.7) | 51.3 (10.1) | 51.9 (9.8) | 57.2 (11.4) | 48.1 (9.5) | 48.6 (6.4) | **<.001** |
| Operation type | Adnex surgery | 14 (24.2%) | 15 (18.1%) | 17 (30.4%) | 24 (36.4%) | 23 (44.2%) | - | - | - | - | - | - | - | - |
|  | Hernia inguinalis (laparoscopic) | 26 (44.8%) | 40 (48.2%) | 22 (39.3%) | 20 (30.3%) | 13 (25.0%) |  | - | - | - | - | - | - |  |
|  | Hernia inguinalis (open) | 0 | 0 | 1 (1.8%) | 1 (1.5%) | 1 (1.9%) |  | - | - | - | - | - | - |  |
|  | Cholecystectomy | 18 (31.0%) | 28 (33.7%) | 16 (28.5%) | 21 (31.8%) | 15 (28.9%) |  | - | - | - | - | - | - |  |
|  | Hysterectomy (laparoscopic) | - | - | - | - | - | - | 20 (30.8%) | 25 (51.0%) | 20 (44.4%) | 14 (22.2%) | 30 (62.5%) | 43 (67.2%) | - |
|  | Hysterectomy (open) | - | - | - | - | - |  | 2 (3.1%) | 6 (12.2%) | 9 (20.0%) | 3 (4.8%) | 10 (20.8%) | 12 (18.8%) |  |
|  | Colectomy (laparoscopic) | - | - | - | - | - |  | 40 (61.5%) | 14 (28.6%) | 13 (28.9%) | 35 (55.5%) | 7 (14.6%) | 8 (12.5%) |  |
|  | Colectomy (open) | - | - | - | - | - |  | 3 (4.6%) | 4 (8.2%) | 3 (6.7%) | 11 (17.5%) | 1 (2.1%) | 1 (1.5%) |  |
| Smoking behaviour | Yes | 6 (10.3%) | 15 (18.1%) | 16 (28.6%) | 12 (18.2%) | 8 (15.4%) | .149 | 4 (6.2%) | 10 (20.4%) | 10 (22.2%) | 7 (11.1%) | 2 (4.2%) | 13 (20.3%) | **.014** |
|  | No | 52 (90.7%) | 68 (81.9%) | 40 (71.4%) | 54 (72.8%) | 44 (84.6%) |  | 61 (93.8%) | 39 (79.6%) | 35 (77.8%) | 56 (88.9%) | 46 (95.8%) | 51 (79.7%) |  |
| Educational level | Low | 3 (5.2%) | 5 (6.0%) | 14 (25.0%) | 2 (3.0%) | 6 (11.5%) | **<.001** | 9 (13.9%) | 4 (8.2%) | 5 (11.1%) | 7 (11.1%) | 2 (4.2%) | 5 (7.8%) | .21 |
|  | Medium | 9 (15.5%) | 40 (48.2%) | 21 (37.5%) | 26 (39.4%) | 29 (55.8%) |  | 26 (40.0%) | 20 (40.8%) | 27 (60.0%) | 33 (52.4%) | 24 (50.0%) | 39 (60.9%) |  |
|  | High | 46 (79.3%) | 38 (45.8%) | 21 (37.5%) | 38 (47.6%) | 17 (32.7%) |  | 30 (46.1%) | 25 (51.0%) | 13 (28.9%) | 23 (36.5%) | 22 (45.8%) | 20 (31.3%) |  |
| Employment type | Paid work | 46 (79.3%) | 62 (74.7%) | 39 (69.6%) | 50 (75.8%) | 37 (71.2%) | .782 | 39 (60.0%) | 37 (75.5%) | 27 (60.0%) | 35 (55.6%) | 40 (83.3%) | 53 (82.8%) | **<.001** |
|  | No paid work | 12 (20.7%) | 21 (25.3%) | 17 (30.4%) | 16 (24.2%) | 15 (28.8%) |  | 26 (40.0%) | 12 (24.5%) | 18 (40.0%) | 28 (44.4%) | 12 (16.7%) | 11 (17.2%) |  |
| Work hours | Median [IQR] | 36.0 [32.0;40.0] | 36.0 [26.5;40.0] | 32.0 [24.0;40.0] | 36.0 [26.5;40.0] | 32.0 [30.0;40.0] | .642 | 35.0 [24.5;40.0] | 36.0 [24.0;40.0] | 32.0 [24.8;37.0] | 32.0 [28.0;40.0] | 32.0 [28.0;40.0] | 32.0 [26.0;38.8] | .596 |
| Work appraisal | Good | 43 (91.5%) | 53 (84.1%) | 27 (69.2%) | 44 (86.3%) | 29 (78.4%) | - | 43 (100.0%) | 28 (75.7%) | 23 (82.1%) | 29 (82.9%) | 32 (80.0%) | 42 (77.8%) | - |
|  | Fair | 4 (8.5%) | 10 (15.9%) | 12 (30.8%) | 4 (7.8%) | 7 (18.9%) |  | 0 | 9 (24.3%) | 4 (14.3%) | 6 (17.1%) | 7 (17.5%) | 10 (18.5%) |  |
|  | Mediocre | 0 | 0 | 0 | 2 (3.9%) | 1 (2.7%) |  | 0 | 0 | 1 (3.6%) | 0 | 1 (2.5%) | 0 |  |
|  | Bad | 0 | 0 | 0 | 1 (2.0%) | 0 |  | 0 | 0 | 0 | 0 | 0 | 2 (3.7%) |  |
|  | NA | 11 | 20 | 17 | 15 | 15 |  | 22 | 12 | 17 | 28 | 8 | 10 |  |
| Expectations for full return to work (RTW) | Median [IQR] | 7.0 [4.0;14.0] | 14.0 [6.0;14.0] | 14.0 [7.0;14.0] | 10.0 [7.0;14.0] | 14.0 [7.0;28.0] | **.003** | 28.0 [24.5;42.0] | 42.0 [28.0;42.0] | 42.0 [35.0;50.8] | 42.0 [31.5;52.5] | 38.5 [28.0;42.0] | 42.0 [29.8;42.0] | **<.001** |
| Expectations for full return to normal activities (RNA) | Median [IQR] | 14.0 [7.0;14.0] | 14.0 [10.0;21.0] | 14.0 [13.5;28.0] | 14.0 [10.0;21.0] | 21.0 [14.0;28.0] | **.004** | 35.0 [28.0;56.0] | 42.0 [28.0;42.0] | 42.0 [42.0;56.0] | 42.0 [28.0;56.0] | 42.0 [28.0;50.8] | 42.0 [42.0;50.8] | .059 |
| Perceived health | Median [IQR] | 90.0 [80.0;95.0] | 80.0 [70.0;85.0] | 66.5 [57.5;76.2] | 85.0 [70.0;90.0] | 75.0 [53.8;85.0] | **<.001** | 86.0 [80.0;90.0] | 80.0 [70.0;85.0] | 60.0 [50.0;75.0] | 80.0 [70.0;90.0] | 80.0 [80.0;90.0] | 69.0 [55.0;80.0] | **<.001** |
| Adjuvant chemotherapy | No | - | - | - | - | - | - | 58 (96.7%) | 48 (100.0%) | 39 (86.7%) | 44 (71.0%) | 47 (100.0%) | 64 (100.0%) | - |
|  | Yes | - | - | - | - | - |  | 2 (3.3%) | 0 | 6 (13.3%) | 18 (29.0%) | 0 | 0 |  |
|  | NA | - | - | - | - | - |  | 5 | 1 | 0 | 1 | 1 | 0 |  |
| Complications | No | 58 (100.0%) | 82 (98.8%) | 55 (98.2%) | 64 (97.0%) | 48 (92.3%) | - | 58 (89.2%) | 45 (91.8%) | 31 (68.9%) | 45 (72.6%) | 41 (85.4%) | 58 (90.6%) | **.002** |
|  | Yes | 0 | 1 (1.2%) | 1 (1.8%) | 2 (3.0%) | 4 (7.7%) |  | 7 (10.8%) | 4 (8.2%) | 14 (31.1%) | 17 (27.4%) | 7 (14.6%) | 6 (9.4%) |  |
|  | NA | 0 | 0 | 0 | 0 | 0 |  | 0 | 0 | 0 | 1 | 0 | 0 |  |
| Difficulty of the selected PROMIS-PF items | Mean (sd) | -0.61 (0.06) | -0.60 (0.07) | -0.61 (0.07) | -0.60 (0.07) | -0.63 (0.06) | .284 | -0.58 (0.06) | -0.59 (0.05) | -0.62 (0.06) | -0.60 (0.06) | -0.61 (0.05) | -0.61 (0.05) | **<.001** |

***** data are presented as the mean and standard deviation for normally distributed variables, the median (interquartile range [IQR]) for skewed variables and frequencies (%) for categorical variables.

** One-way ANOVA for normally distributed variables, Kruskal–Wallis ANOVA for non-normally distributed variables, Chi-square for frequencies.

## **SDC 8:** GMM cluster characteristics

| Variable | Metric* | Clusters | | | | | | | | |
| --- | --- | --- | --- | --- | --- | --- | --- | --- | --- | --- |
|  |  | RCT1 | | | | RCT2 | | | | |
|  |  | A  *Fast*  n=65 (20.6%) | B  *Intermediate*  n=140 (44.4%) | C  *Uneven*  n=110 (34.9%) | p** | A  *Fast*  n=53 (15.9%) | B  *Intermediate*  n=175 (52.4%) | C  *Uneven*  n=92 (27.5%) | D  *Relapse*  n=14 (4.2%) | p** |
| Physical function | Baseline | 59.6 [53.3;61.3] | 53.7 [49.5;59.8] | 45.4 [40.2;51.6] | **<.001** | 52.2 [47.1;60.6] | 52.2 [45.5;59.9] | 46.1 [39.5;51.3] | 57.6 [49.5;59.7] | **<.001** |
|  | 1 week | 43.7 [39.5;45.5] | 42.6 [38.2;50.8] | 37.7 [32.8;43.2] | **<.001** | - | - | - | - | - |
|  | 2 weeks | - | - | - | - | 41.5 [37.1;47.4] | 37.9 [32.6;43.8] | 34.6 [30.5;39.2] | 37.8 [35.4;42.5] | **<.001** |
|  | 3 weeks | 60.3 [58.6;61.0] | 50.5 [46.0;54.8] | 44.1 [39.6;49.4] | **<.001** | - | - | - | - | - |
|  | 4 weeks | - | - | - | - | 46.5 [42.4;50.1] | 44.4 [40.8;50.6] | 38.6 [33.9;44.2] | 45.8 [42.8;48.0] | **<.001** |
|  | 6 weeks | 58.5 [57.8;59.3] | 57.9 [56.7;58.9] | 45.4 [41.7;49.5] | **<.001** | 60.3 [59.2;61.7] | 48.7 [44.0;53.3] | 43.0 [36.8;48.1] | 52.1 [47.6;57.3] | **<.001** |
|  | 12 weeks | 60.6 [59.4;61.3] | 59.8 [58.2;61.0] | 49.6 [44.0;58.1] | **<.001** | 60.5 [59.2;61.7] | 59.0 [52.0;60.5] | 46.4 [39.9;50.3] | 57.6 [48.8;58.7] | **<.001** |
|  | 26 weeks | - | - | - | - | 60.5 [59.2;61.8] | 59.6 [57.7;60.9] | 46.7 [41.5;51.3] | 41.4 [36.7;44.2] | **<.001** |
|  | 52 weeks | - | - | - | - | 60.5 [59.2;61.8] | 59.8 [58.5;61.0] | 45.9 [42.4;49.3] | 58.8 [58.1;60.5] | **<.001** |
| Sex | Male | 33 (50.8%) | 63 (45.0%) | 47 (42.7%) | .582 | 12 (22.6%) | 51 (29.1%) | 29 (31.5%) | 6 (42.9%) | .458 |
|  | Female | 32 (49.2%) | 77 (55.0%) | 63 (57.3%) |  | 41 (77.4%) | 124 (70.9%) | 63 (68.5%) | 8 (57.1%) |  |
| Age | Mean (sd) | 48.9 (13.1) | 50.4 (12.1) | 50.8 (13.4) | .601 | 51.9 (10.2) | 52.2 (10.1) | 54.6 (10.7) | 49.0 (12.5) | .128 |
| Operation type | Adnex surgery | 14 (21.5%) | 42 (30.0%) | 37 (33.6%) | - | - | - | - | - | - |
|  | Hernia inguinalis (laparoscopic) | 32 (49.2%) | 47 (33.6%) | 42 (38.2%) |  | - | - | - | - |  |
|  | Hernia inguinalis (open) | 0 | 2 (1.4%) | 1 (0.9%) |  | - | - | - | - |  |
|  | Cholecystectomy | 19 (29.2%) | 49 (35.0%) | 30 (27.3%) |  | - | - | - | - |  |
|  | Hysterectomy (laparoscopic) | - | - | - |  | 29 (54.7%) | 88 (50.3%) | 32 (34.8%) | 3 (21.4%) | - |
|  | Hysterectomy (open) | - | - | - |  | 7 (13.2%) | 20 (11.4%) | 13 (14.1%) | 2 (14.3%) |  |
|  | Colectomy (laparoscopic) | - | - | - |  | 15 (28.3%) | 58 (33.1%) | 35 (38.1%) | 9 (65.3%) |  |
|  | Colectomy (open) | - | - | - |  | 2 (3.8%) | 9 (5.2%) | 12 (13.0%) | 0 |  |
| Smoking behaviour | Yes | 12 (18.5%) | 22 (15.7%) | 23 (20.9%) | .569 | 7 (13.2%) | 20 (11.4%) | 17 (18.5%) | 2 (14.3%) | .467 |
|  | No | 53 (81.5%) | 118 (84.3%) | 87 (79.1%) |  | 46 (86.8%) | 155 (88.6%) | 75 (81.5%) | 12 (85.7%) |  |
| Educational level | Low | 3 (4.6%) | 10 (7.1%) | 17 (15.5%) | **.003** | 4 (7.6%) | 17 (9.7%) | 11 (12.0%) | 0 | - |
|  | Medium | 21 (32.3%) | 52 (37.1%) | 52 (47.3%) |  | 23 (43.4%) | 86 (49.2%) | 50 (54.3%) | 10 (71.4%) |  |
|  | High | 41 (63.1%) | 78 (55.8%) | 41 (37.2%) |  | 26 (49.0%) | 72 (41.1%) | 31 (33.7%) | 4 (28.6%) |  |
| Work type | Paid work | 47 (72.3%) | 108 (77.1%) | 79 (71.8%) | .582 | 40 (75.5%) | 128 (73.1%) | 52 (56.5%) | 11 (78.6%) | **.021** |
|  | No paid work | 18 (27.7%) | 32 (22.9%) | 31 (28.2%) |  | 13 (24.5%) | 47 (27.9%) | 40 (43.5%) | 3 (21.4%) |  |
| Work hours | Median [IQR] | 36.0 [30.0;40.0] | 36.0 [25.0;40.0] | 32.0 [24.0;40.0] | .235 | 32.0 [24.0;39.0] | 32.0 [27.2;40.0] | 32.0 [29.0;40.0] | 30.0 [24.0;38.0] | .528 |
| Work appraisal | Good | 39 (83.0%) | 97 (87.4%) | 60 (76.0%) | - | 38 (88.4%) | 105 (80.8%) | 45 (84.9%) | 9 (81.8%) | - |
|  | Fair | 6 (12.8%) | 13 (11.7%) | 18 (22.8%) |  | 5 (11.6%) | 22 (16.9%) | 7 (13.2%) | 2 (18.2%) |  |
|  | Mediocre | 1 (2.1%) | 1 (0.9%) | 1 (1.2%) |  | 0 | 1 (0.8%) | 1 (1.9%) | 0 |  |
|  | Bad | 1 (2.1%) | 0 | 0 |  | 0 | 2 (1.5%) | 0 | 0 |  |
|  | NA | 18 | 29 | 31 |  | 10 | 45 | 39 | 3 |  |
| Expectations for full return to work | Median [IQR] | 10 [5.0;14.0] | 10 [7.0;14.0] | 14 [7.0;21.0] | .123 | 35.0 [28.0;42.0] | 42.0 [28.0;42.0] | 42.0 [35.0;56.0] | 42.0 [42.0;56.0] | **<.001** |
| Expectations for full recovery of normal activities | Median [IQR] | 14.0 [7.0;21.0] | 14.0 [10.0;28.0] | 14.0 [12.5;28.0] | .133 | 42.0 [28.0;42.0] | 42.0 [28.0;52.5] | 42.0 [28.0;56.0] | 49.0 [42.0;77.0] | **.025** |
| Perceived health | Median [IQR] | 87.0 [80.0;95.0] | 80.0 [70.0;90.0] | 70.1 [56.2;80.0] | **<.001** | 80.0 [75.0;90.0] | 80.0 [65.0;90.0] | 70.0 [50.0;80.0] | 80.0 [50.0;88.8] | **.002** |
| Adjuvant chemotherapy | No | - | - | - | - | 52 (100.0%) | 166 (97.1%) | 74 (82.2%) | 8 (61.5%) | **<.001** |
|  | Yes | - | - | - |  | 0 | 5 (2.9%) | 16 (17.8%) | 5 (38.5%) |  |
|  | NA | - | - | - |  | 48 | 152 | 68 | 10 |  |
| Complications | No | 64 (98.5%) | 136 (97.1%) | 107 (97.3%) | - | 48 (90.5%) | 152 (86.9%) | 68 (74.7%) | 10 (71.4% ) | - |
|  | Yes | 1 (1.5%) | 4 (2.9%) | 3 (2.7%) |  | 5 (9.5%) | 23 (13.1%) | 23 (25.3%) | 4 (28.6%) |  |
|  | NA | - | - | - |  | 0 | 0 | 1 | 0 |  |
| Difficulty of the selected PROMIS-PF items | Mean (sd) | -0.61 (0.05) | -0.61 (0.07) | -0.61 (0.07) | .978 | -0.59 (0.06) | -0.60 (0.06) | -0.61 (0.05) | -0.63 (0.05) | **.029** |

***** data are presented as the mean and standard deviation for normally distributed variables, the median (interquartile range [IQR]) for skewed variables and frequencies (%) for categorical variables.

** One-way ANOVA for normally distributed variables, Kruskal–Wallis ANOVA for non-normally distributed variables, Chi-square for frequencies..
